# Cefdinir reprograms Gram‐positive bacteria to synergize with lysozyme against superbugs

**DOI:** 10.1002/mlf2.70080

**Published:** 2026-06-12

**Authors:** Qi Zhang, Yang Yang, Shuqi Li, Zhao Liu, Chun‐Kit Lee, Chi‐Bun Ko, Qian Zhao

**Affiliations:** ^1^ State Key Laboratory of Chemical Biology and Drug Discovery, Department of Applied Biology and Chemical Technology The Hong Kong Polytechnic University Hong Kong China; ^2^ Centre for Eye and Vision Research (CEVR), Hong Kong Science Park Hong Kong China

**Keywords:** antimicrobial resistance, cefdinir, lipoteichoic acid, lysozyme, wall teichoic acid

## Abstract

Multidrug‐resistant (MDR) bacteria pose a critical global health threat, urgently requiring solutions. Cefdinir, the highest‐selling third‐generation cephalosporin but one now facing clinical obsolescence due to escalating resistance, is conventionally classified as a nascent cell wall synthesis inhibitor. This study challenges this understanding by demonstrating that cefdinir renders existing cell walls negatively charged, thereby synergizing with ubiquitous lysozyme and simultaneously restoring both agents' efficacy against a broad spectrum of MDR Gram‐positive superbugs without driving resistance. Specifically, cefdinir is unexpectedly shown to post‐transcriptionally increase the levels of clustered enzymes in lipoteichoic acid (LTA) and wall teichoic acid (WTA) synthesis pathways, including TarA, TarB, TarD, TarF, TarH, TarK, TarL, TarS, and FmtA, thereby enhancing cell wall electronegativity to remodel existing cell wall into a lysozyme‐susceptible state, in which TarS is identified for the first time by mass spectrometry. The combination of cefdinir and lysozyme also significantly suppresses biofilm formation and minimizes *de novo* resistance mutations. Antibacterial efficacy of combination therapy is validated in both cell‐based infection models and rat skin infection models, demonstrating strong translational potential. Given the established safety profiles of both agents, this sensitization strategy can be applied to human clinical research to combat MDR Gram‐positive bacterial infections.

## Impact statement

Antimicrobial resistance (AMR) threatens patient safety and worsens medical experience, especially when involving widely used drugs like the highest‐selling cefdinir and ubiquitous lysozyme. While enhancing intrinsic efficacy of the drugs is a conventional strategy, its limitations have become apparent. Here, we discover a novel function of cefdinir in rendering the existing cell wall negatively charged. This novel mechanism enables cefdinir to synergize with the natural defense lysozyme (through extrinsic activation via charge manipulation), restoring both agents' efficacy against broad‐spectrum MDR Gram‐positive superbugs without driving resistance. The cefdinir and lysozyme combination demonstrates *in vivo* efficacy and biofilm suppression capability, highlighting its potential for clinical translation.

## INTRODUCTION

Since the introduction of penicillin in the 1940s, antimicrobial resistance (AMR) has consistently posed a global threat^1^. In order to raise awareness within the scientific community, the World Health Organization (WHO) has identified 12 families of pathogens exhibiting high resistance levels worldwide. Unfortunately, a large proportion of bacterial infections, particularly those acquired in communities and hospitals^2^, are linked to these pathogens^3^. Among them, methicillin‐resistant *Staphylococcus aureus* (MRSA)^4^ and methicillin‐resistant *Staphylococcus epidermidis* (MRSE)^5^ stand out as the two most notorious superbugs, particularly in ocular and skin bacterial infections^6^, ^7^, ^8^. An existing pathogenic example is contagious bacterial conjunctivitis, causing a rising incidence of blindness^9^, ^10^, in which MRSA (one of the primary pathogens) becomes increasingly difficult to treat. Moreover, continuous exposure to medical devices, such as lenses, facilitates biofilm formation by superbugs, further compromising treatment efficacy^11^. These pathogenic cases have rendered an increasing number of clinical drugs ineffective, further worsening patients' satisfaction with medical treatment. While potent last‐line agents like vancomycin and linezolid remain the current standard of care for these infections, the relentless evolution of resistance mechanisms threatens their long‐term efficacy and underscores the precarious reality of the dawning postantibiotic era. The alarming rise of multidrug‐resistant pathogens signifies that today's effective therapeutics may become obsolete tomorrow. This imminent threat constitutes a fundamental driver for the global AMR research community, which is urgently exploring proactive strategies and alternative therapeutic options to future‐proof our clinical arsenal. Among them, one pressing need is to address superbugs resistant to top‐prescribed drugs, primarily due to the lack of practical alternatives with substantial market demand in clinical settings in the short term.

Cefdinir, a widely recognized third‐generation oral cephalosporin, has emerged as the highest‐selling antimicrobial (e.g., up to $585 million in the United States in 2008^12^) due to its broad‐spectrum activity against both Gram‐positive and Gram‐negative pathogens. Its classical mechanism of action involves inhibiting nascent bacterial cell wall synthesis by binding to penicillin‐binding proteins (PBPs)^13^, although variants such as PBP2a exhibiting lower binding affinity^14^, ^15^, confer resistance. Lysozyme, one of the most widely used natural defenses present in most living organisms (over 1 mg/ml in tears for adults^16^), can eliminate Gram‐positive bacteria by hydrolyzing 1,4‐β‐linkages between N‐acetyl‐d‐glucosamine and N‐acetylmuramic acid in bacterial peptidoglycan, a layer that is thick and accessible in Gram‐positive bacteria while being thin and protected by an additional outer membrane in Gram‐negative bacteria^17^. Therefore, it continuously alleviate the burden on traditional antimicrobials by daily sharing their antimicrobial workload. Despite their mechanistic distinctions, both cefdinir and lysozyme are facing clinical obsolescence due to escalating resistance^16^, ^17^. Current research on these agents primarily focuses on structural optimization to combat resistance^17^, whereas their studies investigating additional functional insights or combination therapies remain limited.

Combination therapy composed of an antimicrobial and its adjuvant (acting as a resistance breaker) has emerged as a safe, economical, and effective alternative due to its efficacy in reducing the clinical dosage of antimicrobials and slowing the emergence of MDR superbugs^18^, ^19^, ^20^, ^21^. Meanwhile, the back‐to‐basics strategy of repurposing existing drugs, particularly natural metabolites or compounds with known pharmacokinetics and safety profiles, has also gained popularity due to its feasibility for rapid clinical approval^19^, ^20^. A typical example is glutamine, a natural metabolite, repurposed as a resistance breaker to restore ampicillin sensitivity in MDR *Pseudomonas aeruginosa* ^22^.

In this study, through high‐throughput screening, we discovered that “the highest‐selling” cefdinir synergizes with lysozyme, thereby simultaneously restoring both agents' efficacy against broad‐spectrum MDR Gram‐positive superbugs, including MRSA. This synergy stems from a novel mechanism of cefdinir whereby it renders bacterial cell walls negatively charged. Our findings offer a ready‐to‐implement combination therapeutic strategy for immediate clinical evaluation, particularly valuable for resource‐limited settings.

## RESULTS

### High‐throughput screening identifies the synergistic effect between cefdinir and lysozyme

**Figure 1:**
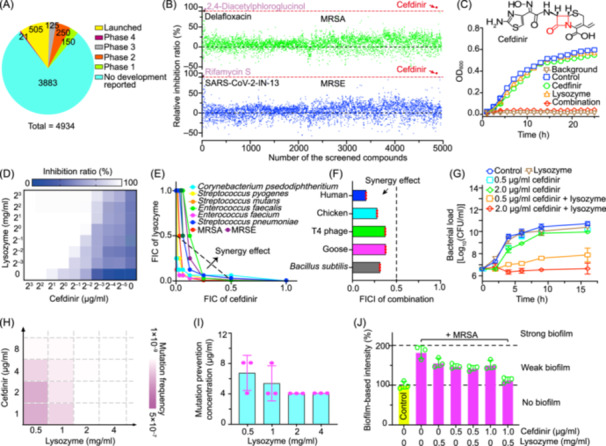
The high‐throughput screening reveals the synergy between cefdinir and lysozyme against a broad spectrum of MDR Gram‐positive bacteria. (A, B) A total of 4934 compounds, consisting of 6 subclasses (A) at fixed concentrations (2.5 μM), tested in the absence or presence of 1 mg/ml human lysozyme against MRSA and MRSE. (B). We tested all compounds in the library without any bias regarding their original intended targets. Although there were other drugs with inhibition ratios greater than 90% (indicated by red dashed lines) in MRSA (2,4‐diacetylphloroglucinol and Delafloxacin) and MRSE (Rifamycin S and SARS‐CoV‐2‐IN‐13), only cefdinir exhibited dual inhibition (indicated by the red point). These clinical development phases of compounds were collected from https://clinicaltrials.gov/ (accessed on October 14, 2025). (C) The real‐time growth curves showing the optical density (OD) of MRSA under different treatments for 22 h. The inset is the structure of cefdinir, in which its core structure is highlighted in a red area. (D) Representative heatmap of MRSA growth inhibition in a checkerboard broth micro‐dilution assay with increasing concentrations of cefdinir and lysozyme. (E) Isobologram illustrating combination therapies against 8 common MDR Gram‐positive bacteria. (F) FICI analysis of combination therapies between cefdinir and 5 subtypes of lysozyme against MRSA. The labels “Human,” “Chick,” “T4 phage,” “Goose,” and *“Bacillus subtilis*” denote the C‐type lysozyme derived from *Homo sapiens*, the C‐type lysozyme derived from *Gallus gallus*, the T4‐type lysozyme derived from T4 phage, the G‐type lysozyme from *Anser anser*, and the bacterial‐type lysozyme from *B. subtilis*, respectively. (G) Time‐killing curves depicting the growth of log‐phase MRSA treated with cefdinir in the absence or presence of 0.5 mg/ml lysozyme for 16 h. That, without any treatment, served as the control group. (H) Heatmap of bacterial mutant frequencies in MRSA treated with increasing bactericidal concentrations of the cefdinir and lysozyme combination. The mutant frequency was determined by counting surviving colonies relative to the initial colony‐forming units. (I) Scatter plot showing the mutation prevention concentration of cefdinir against MRSA under different lysozyme concentrations, with mutation prevention concentration values ranging from 4 to 8 μg/ml. (J) Crystal violet assays demonstrating the effect of lysozyme, cefdinir, or their combination on MRSA pre‑biofilm formation. All assays were performed in triplicate and results were expressed as mean ± SD unless otherwise stated. FICI, fractional inhibitory concentration index; MDR, multidrug‐resistant; FIC, fractional inhibitory concentration; MRSA, methicillin‐resistant *Staphylococcus aureu*s; MRSE, methicillin‐resistant *Staphylococcus epidermidis*.

Using MRSA (USA300, ATCC BAA‐1556) and MRSE (ATCC 35984) as two model strains, which are representative pathogens in hospital‐acquired infections^6^, ^7^, ^8^, we conducted a large‐scale screening of 4934 compounds across six sub‐classes (fixed at 2.5 μM) to assess whether the activity of lysozyme, known as the most widely used natural defender, can be restored by monitoring bacterial growth curves over a period of 22 h (Figures 1A–C, S1 and Table S1). The primary screening identified several hits that exhibited enhanced inhibitory activities in combination. Among these hits, cefdinir, the highest‐selling third‐generation cephalosporin^23^, was observed to have synergistic effects in combination with 1 mg/ml lysozyme, achieving ≥90% combination inhibition ratios against both MRSA and MRSE (Table S1). Cefdinir is traditionally regarded as primarily targeting PBPs to inhibit cell wall synthesis, and is widely used to treat ENT (ear, nose, and throat) and skin infections. Meanwhile, neither lysozyme nor cefdinir alone showed such inhibitory activity, indicating that the two model strains should be classified as pathogens that are resistant to both lysozyme and cefdinir according to the guidelines of the Clinical and Laboratory Standards Institute (CLSI)^24^ (Figures 1D, S2 and Table S1).

Then, we conducted a standard checkerboard broth microdilution assay in Luria‐Bertani (LB) medium to evaluate this combination against eight common Gram‐positive superbugs, including MRSA, MRSE, *Streptococcus pneumoniae*, *Streptococcus pyogenes*, *Streptococcus mutans*, *Corynebacterium pseudodiphtheriticum*, vancomycin‐resistant *Enterococcus faecium*, and vancomycin‐resistant *Enterococcus faecalis* (Figures 1E and S2). Their synergistic effects were observed in all tested strains, as indicated by the fractional inhibitory concentration index (FICI) values ranging from 0.094 to 0.375 (FICI ≤ 0.5 is defined as synergism, Figure 1E). This phenomenon was also found between cefdinir and another subtype of lysozyme, that is, C‐type (from human and chicken), G‐type (from goose), T4‐type (from T4 phage), and bacterial‐type (from *Bacillus subtilis*) (Figures 1F, S3‐S4 and Table S2). For instance, cefdinir at a concentration of ca. 1 µg/ml (equivalent to 2.5 µM) could re‐sensitize MRSA to human lysozyme, as judged by ≥1200‐fold reduction in the minimum inhibitory concentration (MIC) value of lysozyme from ≥150 to 0.125 mg/ml (Figure 1D). Due to its significance and efficacy, the combination therapy between cefdinir and human lysozyme against MRSA was selected as the representative in subsequent studies.

Next, we conducted the time‐killing analysis^20^ and observed that neither 0.5 mg/ml lysozyme nor 0.5 µg/ml cefdinir (nor even 2.0 µg/ml cefdinir) could inhibit the growth of MRSA during its exponential phase. In contrast, their combination successfully reduced the bacterial load from levels of 10^10^ to 10^7^ colony‐forming units per milliliter (CFU/ml) after 16 h (over 1000‐fold reduction in bacterial load) (Figures 1G and S5A). Notably, the final bacterial concentration fell within or below the standard starting inoculum used in such assays. According to the CLSI standard^24^, a reduction of this magnitude is classified as indicative of bacteriostatic or even bactericidal activity^25^. Given that bacteriostatic agents are clinically non‐inferior to bactericidals in multiple infections, our combination strategy achieves a clinically relevant and potent suppression of bacterial growth.

Considering resistance‐development as a significant challenge in clinical treatment^19^, ^20^, we then assessed the effect of combination therapy on the frequency of resistance mutations. Our results showed that the combination therapy did not increase bacterial mutation frequency, and the prevention concentrations of cefdinir ranged between 4 and 8 µg/ml when used in combination therapies (Figure 1H,I). These findings suggest that lysozyme and cefdinir combination therapy could effectively minimize the *de novo* emergence of resistance development.

Notably, bacterial biofilm often causes ineffectiveness of antimicrobial in clinical treatments^26^, especially for skin and ocular infections^11^, ^27^. We thus conducted crystal violet assays on MRSA at the pre‐biofilm and post‐biofilm stages to determine whether these biofilms could be removed by combination therapy. Excitingly, we observed that 1.0 μg/ml cefdinir and 0.5 mg/ml lysozyme combination therapy significantly decreased the biofilm‐based absorbance to a level similar to the control group, indicating that biofilm had been fully removed under combination therapy (Figure 1J). This reduction in combination therapy was also observed even after the biofilm had formed (Figure S5B). In contrast, monotherapies only caused slight reductions.

### Dual roles of cefdinir in inhibiting nascent cell wall synthesis and re‐sensitizing existing cell walls to lysozyme

**Figure 2:**
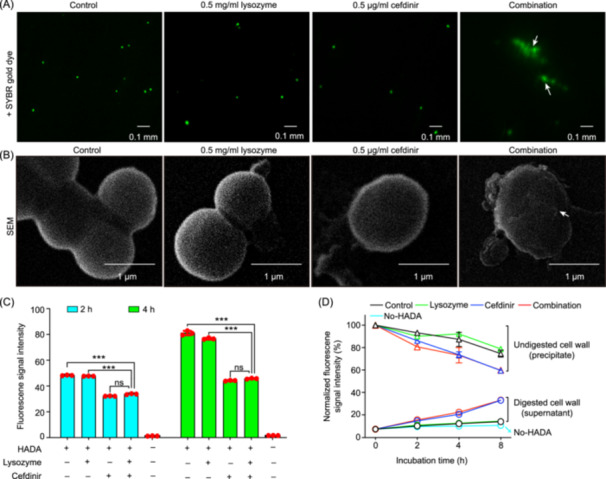
Combination therapy inhibits cell wall synthesis and accelerates lysozyme‐mediated digestion of MRSA. (A, B) Permeability (A) and morphological (B) analyses of MRSA under different treatments. White arrowheads in diffused SYBR gold dye and SEM images of MRSA under combination therapies indicate damaged cell walls with numerous pits and holes. Scale bars, 0.1 mm (A) and 1 μm (B). (C) The HADA‐based synthesis analysis of nascent cell walls of MRSA in the presence of 0.5 mg/ml lysozyme, 0.5 μg/ml cefdinir, or their combination for 2 or 4 h treatment. Fluorescence signal intensity was recorded as RLU at an emission wavelength of 461 nm. Bacteria with and without 100 μM HADA treatment served as the control and no‐drug groups, respectively. Statistical comparisons between two groups were performed using unpaired two‐tailed Student's *t*‐test. ***p < 0.001; ns, not significant. (D) Digestion rate of MRSA cell walls under different treatments. More HADA‐based signals from digested supernatants indicate a greater susceptibility of the existing cell walls to lysozyme. All assays were performed in triplicate and results were expressed as mean ± SD unless otherwise stated. HADA, HCC‐amino‐D‐alanine; RLU, relative light unit; SEM, scanning electron microscope.

To investigate the mechanism underlying the synergistic effect between cefdinir and lysozyme, we first conducted a permeability analysis on MRSA pre‐treated with cefdinir (0.5 μg/ml) in the absence or presence of lysozyme (0.5 mg/ml). Propidium iodide (PI), a red‐fluorescent DNA dye that can only penetrate the plasma membrane of nonviable cells^28^, showed no fluorescence in any treatment groups (Figure S6A). In contrast, when nucleic acid fluorochrome SYBR gold^29^ was introduced into an MRSA‐agarose mixture to test whether the bacterial cell wall was damaged, the nucleic acid diffusion was clearly observed in the combination group but not in the other groups (Figure 2A). These results indicated that the cell wall, rather than the cell membrane, was significantly damaged under the combined treatment.

Given that both lysozyme^17^ and cefdinir^23^ are known to affect bacterial cell walls, we then conducted the scanning electron microscope (SEM) imaging analysis^5^ to directly observe the morphological changes in MRSA under different treatments. As shown in the SEM images in Figure 2B, the cell surface in the untreated group was relatively smooth. Meanwhile, treatments with either cefdinir (0.5 μg/ml) or lysozyme (0.5 mg/ml) alone had minimal effects on bacterial morphology. Interestingly, the bacterium under combination treatment had a distinct digestion‐like roughness in its cell wall (Figure 2B). Predictably, the damage was so severe that it was hardly able to self‐repair and survive.

Next, we investigated whether the observed digestion‐like roughness in the cell wall was the result of continuous digestion of the existing cell wall or the defective synthesis of the nascent cell wall. To do it, we used HCC‐amino‐d‐alanine (HADA)^30^, an analog of d‐alanine found in nascent cell walls, to assess the inhibition effects on cell wall synthesis under different treatments. We observed ca. 40% reduction in HADA signal intensity in MRSA under 2‐h treatment with cefdinir, regardless of whether lysozyme was added or not (Figure 2C). The decrease would be more pronounced if the bacteria were treated for an extended period. Such phenomenon should attribute to the ability of cefdinir as β‐lactam antimicrobial to inhibit the synthesis of nascent cell wall^23^. Unexpectedly, after normalization of nascent cell walls from sonicated bacteria, we observed that digestion with sufficient freshly added lysozyme resulted in a faster release rate of HADA into the supernatant when the cell walls were separated by centrifugation from bacteria that had been treated with cefdinir monotherapy or combination therapy, compared to other treatments, indicating that cefdinir accelerated the digestion of nascent cell walls (Figure 2D). However, we did not observe this difference in MRSA treated with other β‐lactam antimicrobials like cefazolin or cefalexin (Figure S6B,C), implying that this sensitization might be attributed to cefdinir itself rather than to its general effects as a β‐lactam antimicrobial, although more evidence would be required to definitively establish this specificity. In conclusion, cefdinir's traditional view as an antimicrobial was challenged, as it not only inhibited nascent cell wall synthesis but also increased bacterial susceptibility to lysozyme (as an adjuvant), leading to irreversible digestion and bacterial death.

### Cell wall electronegativity enhancement via wall teichoic acid (WTA)/lipoteichoic acid (LTA) upregulation confers lysozyme susceptibility

**Figure 3:**
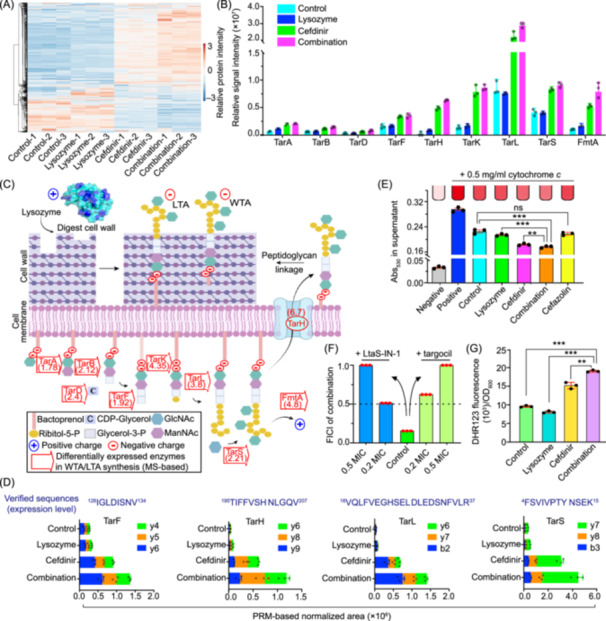
Upregulation of LTA/WTA synthesis induces a lysozyme‐susceptible state in the cell wall, resulting in the re‐sensitization of MRSA to lysozyme. (A) Heatmap displaying DEPs in MRSA under different treatments for 3 h, as determined by TMT‐based quantitative proteomic analysis. (B) Differential expression of selected enzymes involved in the LTA/WTA synthesis pathway. (C) Diagram of the LTA/WTA synthesis pathway in MRSA, with enzymes marked by red arrowheads. Inset data show fold changes in enzyme activity under combination therapy versus lysozyme alone. Lysozyme cartoon (PDB ID: 133 l) was generated using PyMOL. (D) PRM‐based verification of the increased levels of the key enzymes (i.e., TarF, TarH, TarL, and TarS) using unique peptides (shown in insets). (E) Cytochrome *c* binding analysis on MRSA under different treatments. Upper panel, colored schematic after cytochrome *c* treatment; lower panel, reduced absorbance at 530 nm (Abs_530_) under various conditions. Herein, no cytochrome *c* and cytochrome *c* only served as negative and positive groups, respectively. Besides, there are also untreated bacteria (control), and bacteria treated with lysozyme, cefdinir, their combination, or cefazolin (a β‑lactam of the same class as cefdinir). Reduced intensity was observed in untreated bacteria compared to the positive group, indicating that negatively charged bacteria bind to positively charged cytochrome *c*, reducing the amount of free cytochrome *c*. Cefdinir alone or in combination further reduced intensity, whereas lysozyme or cefazolin yielded similar intensity to untreated bacteria. This indicates that cefdinir is the main reason for the enhanced cell‑wall negative charge and interaction with lysozyme. (F) FICI analysis of combination therapy on MRSA with or without treatment of LTA synthesis inhibitor (LtaS‐IN‐1) or WTA synthesis inhibitor (targocil) for 22 h. The increased FICI values indicated that LTA/WTA synthesis played a critical role in the efficacy of combination therapy. (G) ROS levels determined by DHR123 fluorescence in MRSA under different treatments for 1 h. All assays were performed in triplicate and results were expressed as mean ± SD unless otherwise stated. Statistical comparisons between two groups were performed using unpaired two‐tailed Student's *t*‐test. **p < 0.01; ***p < 0.001; and ns, not significant. DEPs, differentially expressed proteins; LTA, lipoteichoic acid; PRM, parallel reaction monitoring; ROS, reactive oxygen species; WTA, wall teichoic acid; MIC, minimum inhibitory concentration; TMT, tandem mass tag.

Considering that acetylation modification in the cell wall has been reported as the main cause of bacterial resistance against lysozyme^16^, we therefore performed an acetylation modification analysis but ultimately ruled out its influence (Figure S7). To further investigate the possible mechanism, we next conducted a proteomic study to identify changes in protein levels in MRSA under different treatments for 3 h. By performing the tandem mass tag (TMT) labeling quantitative proteomic analysis, we found a high degree of similarity in the heatmap depicting differentially expressed proteins (DEPs) between cefdinir monotherapy and combination groups. This finding suggested that the observed combination effect was primarily attributable to cefdinir (Figure 3A). Meanwhile, compared to bacteria treated with lysozyme alone, those undergoing combination therapy exhibited the significant upregulation of 407 DEPs and downregulation of 134 DEPs (Figure S8A). Similar results were also found in bacteria between the cefdinir alone group and the control group (Figures S8–S10). Among these shared changes, we unexpectedly observed the overexpression of clustered enzymes, including TarA, TarB, TarD, TarF, TarH, TarK, TarL, TarS, and FmtA, involved in the synthesis of WTA and LTA (Figure 3B,C). Among these enzymes, TarS, to our knowledge, is first reported as existing evidence in mass spectrometry in MRSA^31^. To confirm these changes in protein levels, we further performed a targeted mass spectrometry analysis known as parallel reaction monitoring (PRM). These results showed trends similar to those observed in the proteomic analysis described above (Figure 3D and Table S3). Interestingly, it was observed that except for *fmtA*, other genes encoding these enzymes were closely clustered in two gene clusters (*tarDBHA* and *tarSLFK* clusters, Figure S11A), suggesting the possibility of cluster‐based induction for general overexpression. To validate it and explore whether their overexpression occurred at the transcriptional levels or not, we next compared their mRNA levels in MRSA under different treatments. Our real‐time quantitative polymerase chain reaction (RT‐qPCR) results indicated that there was a limited change in mRNA levels regardless of how these bacteria were treated (Figure S11B,C). Thus, this cluster‐based overexpression of critical enzymes might be attributed to enhanced efficiency at post‐transcriptional levels (e.g., translation) rather than transcription. Notably, WTA and LTA playe dominant roles in regulating the negative charge of bacterial cell walls^32^, ^33^. Thus, we supposed that increased syntheses of WTA/LTA in cell walls would strengthen the interaction between negatively charged cell walls and positively charged lysozyme.

To further validate the hypothesis, we subsequently conducted a cytochrome *c* binding assay on MRSA under different treatments. Cytochrome *c*, a colorful protein with positive charge in physiological conditions, can bind to bacterial cell walls^34^, ^35^. By incubating 0.5 mg/ml of free cytochrome *c* with MRSA at 37°C for 10 min, we found that treatment with cefdinir instead of cefazolin (a β‐lactam antimicrobial of the same class) resulted in a reduction in free cytochrome *c*, indicating that it specifically led to a more negative charge in cell walls, and thereby caused stronger interaction with positively charged cytochrome *c* (Figure 3E). Thus, it is plausible that the positively charged lysozyme would also bind strongly to the cell walls and subsequently digest them^36^. To test it, the combination effect on MRSA was further evaluated in the presence of inhibitors targeting LTA/WTA synthesis. As shown in Figures 3F and S12, we found that both the LTA synthesis inhibitor (LtaS‐IN‐1) and the WTA synthesis inhibitor (targocil) abolished the synergistic effect, as evidenced by increased FICI values in combination therapies. This suggested that the enhanced binding between the bacterial cell wall and lysozyme indicated in the cytochrome *c* assay was due to increased LTA/WTA synthesis, leading to more susceptible cell walls for lysozyme digestion.

To further understand the potent antimicrobial effect of the combination, we investigated its impact on bacterial physiology. As shown in Figure 3G, we found that this sensitization process also triggered a significant accumulation of intracellular reactive oxygen species (ROS) in MRSA. ROS can induce widespread damage to bacterial cells by oxidizing proteins, lipids, and DNA, thereby contributing to the enhanced killing^37^. In this context, metabolic perturbations, including an altered NAD⁺/NADH ratio and elevated ATP levels, occurred (Figure S13), indicative of metabolic stress. The rise in ROS, potentially linked to this metabolic state, likely acts synergistically with the physical disruption of the cell wall by lysozyme to amplify the antimicrobial outcome.

Taken together, integrating the observed alterations in cell wall biosynthesis with concomitant shifts in metabolic state, our data demonstrated that cefdinir reprograms Gram‐positive bacteria to synergize with lysozyme against drug‐resistant superbugs.

### Combination therapy alleviates infection symptoms and shows translational potential in two infection models

**Figure 4:**
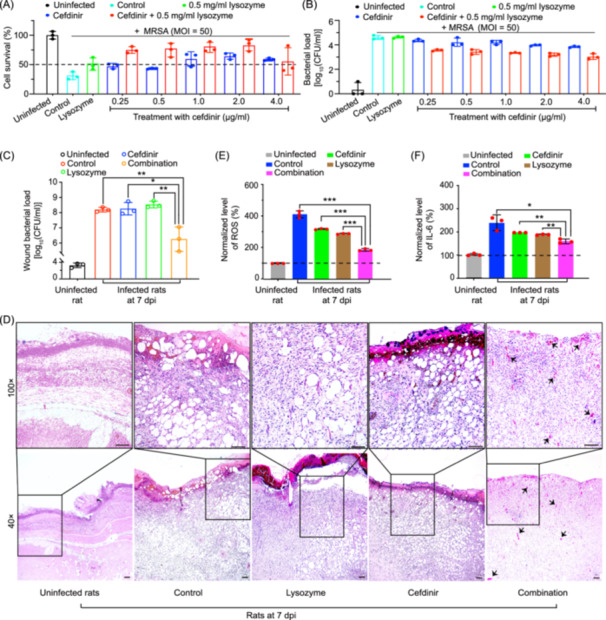
Combination therapy demonstrates an excellent therapeutic effect in both cell‐based and rat skin infection models. (A, B) Survival ratios (A) and bacterial loads (B) of ARPE‐19 cells under different treatments in the cell‐based infection model. The lower survival ratio of the last group might be partly due to the adverse effects of the high‐dose treatment. Bacterial infections were performed on ARPE‐19 cells using the mid‐log‐phase MRSA at an MOI of 50. (C) Bacterial load in the wounds of uninfected or MRSA‑infected rats at 7 dpi under different treatments (untreated infected control, lysozyme alone, cefdinir alone, and their combination). (D) Representative H&E staining images of wounds. Black arrowheads clearly show reduced dense inflammatory cells with blue nuclei, increased new blood vessels, less adipocytes (dissolved to be bubble‐like), and smoother wound edges in the combination therapy group. Scale bars, 0.1 mm. (E, F) Normalized levels of ROS (E) and IL‐6 (F) in the wounds of rats under different treatments. Statistical comparisons between two groups were performed using unpaired two‐tailed Student's *t*‐test. *p < 0.05; **p < 0.01; and ***p < 0.001. dpi, days post inoculation; H&E, hematoxylin and eosin; IL‐6, interleukin 6; MOI, multiplicity of infection.

We further evaluated the synergistic effect using a cell‐based infection model. Given that lysozyme is abundant in tears, we thus selected the ARPE‐19 cell, a spontaneously arising retinal pigment epithelial cell line, as the host cell for the infection assay. We found that neither cefdinir (≤64 μg/ml) nor lysozyme (≤3200 μg/ml) affected the survival ratios of uninfected ARPE‐19 cells (Figure S14); similar conclusions were drawn for epithelial‐like 293 T cells. However, when these cells were infected with MRSA at a multiplicity of infection (MOI) of 50, approximately 80% of infected cells died after 24 h. Interestingly, with the increasing concentration of cefdinir (up to 2 µg/ml) in the presence of 0.5 mg/ml lysozyme, the survival ratio of infected cells increased, and the bacterial load decreased (Figure 4A,B). Specifically, the bacterial load significantly decreased to 3.5 × 10^3^ CFU/ml (3.125%) when 0.25 μg/ml cefdinir was combined with 0.5 mg/ml lysozyme. This level was maintained throughout the entire analyzed period (Figure 4B). These findings demonstrated that the combination effect observed *in vitro* could be successfully translated into the cell‐based infection model.

Given the widespread application of both cefdinir and lysozyme in treating superficial bacterial infections, especially skin infections^16^, ^17^, we next used a rat skin infection model to investigate whether the excellent combination effect could also be replicated *in vivo* (Figure S15A). Specifically, 1.25 ml of 5.0 × 10^11^ CFUs MRSA suspension was sprayed on the skin wound (3 cm × 3 cm) of each anesthetized rat, followed by treatment with sterile medical gauze soaked in normal saline (for control and cefdinir only groups) or lysozyme (70 mg/kg, for lysozyme only and combination groups). Meanwhile, rats in the cefdinir‐only and combination groups further received oral administration (25 mg/kg cefdinir). At 7 days post inoculation (dpi), all rats were sacrificed for the purpose of homogenizing or slicing the wounds. We found that the bacterial load in the wounds of rats undergoing combination therapy was significantly reduced compared to those in the control and monotherapy groups (Figure 4C). Importantly, the remaining bacterial burden (~10^6^ CFU/ml) in combination groups falls within or below the normal range of the minimal infective dose (MID) required to overcome innate host defenses and establish an effective infection in murine models^38^, ^39^. Meanwhile, their inflammatory symptoms were also effectively alleviated, as evidenced by hematoxylin and eosin (H&E) staining analysis. As shown in Figure 4D, denser inflammatory cells with blue nuclei were observed in the control, cefdinir, and lysozyme groups. In contrast, these cells were scarce in the combination therapy group. Besides, more new blood vessels and fewer adipocytes were clearly found in the wound of rats under combination therapy, indicating enhanced tissue regeneration^40^ and reduced inflammatory symptoms^41^. This was further supported by the presence of more pronounced scars and smoother wound edges in combination therapy group (Figures 4D and S15B). Consistent with these findings, there was a significant decrease in the levels of two inflammatory indices, that is, ROS (<65%) and IL‐6 (interleukin 6, <85%) in the wound of rats under combination therapy, if compared with those receiving other treatments (Figure 4E,F). In conclusion, our results demonstrated that combination therapy reduced bacterial load and alleviated infection symptoms, and showed its potential for future clinical application.

## DISCUSSION

Natural evolution enables multicellular organisms, including humans, to thrive in the presence of microbes^42^, ^43^. Tissues like the human eye can be naturally equipped to fend off pathogenic infections through the secretion of various conserved antimicrobials with high biocompatibility^1^, ^44^. Among these, lysozyme stands out as a prominent defender. As a universally present antimicrobial enzyme found in most living organisms, lysozyme has a century‐long history of safety in combating bacterial infections in clinical practice due to its unparalleled advantages, including convenient accessibility, a favorable safety and biocompatibility profile, and its role as the most common self‐applied defense mechanism that operates unconsciously on a daily basis^5^. Furthermore, lysozyme's mature industrial applications, ranging from food preservation and pharmaceuticals to biotechnology and animal husbandry, highlight its scalable production and adaptable potential as a protein that can be engineered and modified for diverse uses. This bridges research in proteomics, industrial production, microbiology, and clinical medicine, particularly in the field of ophthalmology. However, in the context of the predominance of traditional antimicrobials in clinical settings and the ineffectiveness of lysozyme against superbugs, its antibacterial application in conventional therapeutic options is limited. What is worse, due to inaccessible alternatives with a substantial market in the short term, superbugs resistant to widely used drugs have posed a significant health threat to the public^45^, ^46^. Restoring lysozyme activity is significant for reshaping the pharmaceutical industry, as it can share the antimicrobial workload, thereby alleviating the burden on traditional antimicrobials, and also potentiate diverse lysozyme‐based therapeutic efficacies (synergistic effects), paving the way for more sustainable and personalized therapeutic interventions.

Unlike most lysozyme studies focusing on its intrinsic activity enhancement, our research uniquely exploits its extrinsic activation by charge manipulation, circumventing the evolutionary constraints on lysozyme optimization. Specifically, we challenge the conventional view of antimicrobial adjuvants by revealing that cefdinir, traditionally known as a third‐generation cephalosporin inhibiting nascent cell wall synthesis, remodels these existing cell walls into a lysozyme‐susceptible cell wall state in a broad spectrum of MDR Gram‐positive bacteria. By utilizing the most widely used lysozyme and the highest‐selling cefdinir, we synergistically restored the efficacy of both mainstream drugs against broad‐spectrum MDR bacteria. Notably, the presence of the outer membrane in Gram‐negative bacteria constitutes a major barrier to lysozyme penetration. In this context, the synergistic mechanism, wherein cefdinir increases cell wall electronegativity and teichoic acid exposure, is inherently most impactful in Gram‐positive bacteria, where the thick, accessible peptidoglycan layer serves as the primary target for lysozyme.

Notably, their excellent combination efficacy was also well‐translated *in vivo*. The reduction in bacterial load to approximately 10^6 ^CFU/ml, a level at or below the normal range of the MID for murine models, signifies a diminution of the infectious threat^38^, ^39^. This reduction can shift the balance decisively in favor of host immune clearance mechanisms, a principle that underpins the clinical utility of many bacteriostatic antibiotics like tetracyclines in the treatment of acne vulgaris^47^. Notably, the bacterial levels in our murine skin model, consistent with established efficacy benchmarks, may be partly attributed to the presence of the native skin microbiome, which contributes to a naturally high background bacterial load^48^, ^49^. Taken together, this suppression of bacterial growth demonstrates a therapeutic potential. Given the favorable safety profiles of both agents and the well‐characterized protein engineering platforms for lysozyme, this combination could be immediately applied to human clinical research to combat MDR Gram‐positive bacterial infections and rapidly optimized for future personalized enhancements in stability and delivery, especially in both developing countries and resource‐limited settings.

However, it is important to note that bacterial infections are complex and influenced by various factors, including bacterial virulence^50^, ^51^ and the host's immune modulatory responses^52^. In our skin infection model, neither lysozyme nor cefdinir, at their respective dosages, was able to eradicate the bacteria in the wounds of infected rats. Nevertheless, monotherapies appeared to contribute to the recovery process, as evidenced by significantly reduced inflammatory markers, particularly ROS levels. This phenomenon may be attributed to enhanced immune modulation or reduced bacterial virulence^16^, ^53^, ^54^, although more evidence is required to validate them. Notably, the ROS dynamics under different treatments revealed a more complex scenario. While monotherapy mitigated ROS‐associated inflammation in the host, our *in vitro* proteomic analysis suggested that the bacterial cells themselves experienced significant metabolic stress, particularly under combination therapy. The combination treatment triggered a substantial increase in bacterial ROS, likely originating from an enhanced proton motive force (PMF) following perturbations in NADH metabolism and the arrest of cell wall biosynthesis. To counteract this lethal metabolic stress, bacteria likely upregulated ATP synthase to dissipate the excessive PMF through ATP production. This compensatory mechanism, operating on a background of reduced energy consumption from inhibited cell wall synthesis, resulted in the net increase in ATP levels, while simultaneously helping to mitigate ROS generation. This intricate interplay between metabolic stress and adaptive responses underscores the value of our proteomic profiling.

In this study, by comparing the proteomic profiles of bacteria treated with cefdinir and/or lysozyme, we identified TarS, to our knowledge, for the first time, as the existing evidence in mass spectrometry in this superbug. Another intriguing finding from our proteomic analysis was the coordinated upregulation of multiple synthases involved in WTA and LTA biosynthesis following the combination treatment. Notably, the intracellular level of any protein represents a net balance between its biosynthesis and degradation. Considering the genetic organization of these enzymes into contiguous clusters, this synchronous increase initially suggested a potential enhancement at the transcriptional level, which would tilt this balance toward increased synthesis. However, transcriptional analysis via RT‐qPCR revealed no significant alterations in the corresponding mRNA abundances. This discrepancy points toward a posttranscriptional mode of regulation, potentially driven by mechanisms such as a global enhancement of translational efficiency across the polycistronic mRNA^55^, mediated by structural rearrangements that improve ribosome accessibility^56^, or by a decrease in the rate of protein turnover^57^. We speculate that the combination therapy may enhance either the translation of these synthase mRNAs or the stability of these synthase proteins, possibly by interfering with a dedicated regulatory system. Such post‐transcriptional regulation would lead to protein accumulation without a concomitant increase in mRNA, tipping the net balance toward accumulation and effectively amplifying the flux through the WTA/LTA synthesis pathways. While this hypothesis requires further validation through direct ribosome profiling and protein stability assays to distinguish between these possibilities, it provides a compelling, nontranscriptional explanation for our observations. These insights lay a foundation for understanding the mechanisms of action and the patterns of sensitization.

## MATERIALS AND METHODS

### Animals, cell lines, bacterial strains, and reagents

The bacterial strains used in this study included methicillin‐resistant *S. aureus* USA 300 (MRSA, ATCC BAA‐1556), methicillin‐resistant *S. epidermidis* (MRSE, ATCC 35984), and several others obtained from the China General Microbiological Culture Collection Center (CGMCC): *C. pseudodiphtheriticum* (CGMCC code: 1.592), *S. pyogenes* (CGMCC code: 1.8868), *S. pneumoniae* (CGMCC code: 1.8722), *E. faecalis* (CGMCC code: 1.10682), *E. faecium* (CGMCC code: 1.15321), and *Streptococcus* mutans (CGMCC code: 1.2499). Two human cell lines, ARPE‐19 (ATCC CRL‐2302) and 293T (ATCC CRL‐3216), were also sourced from the American Type Culture Collection (ATCC). Goose eggs (*Anser cygnoides*) were purchased via Etsy. Various lysozymes were acquired from different suppliers: human and chicken C‐type lysozymes from ATCG, T4 phage lysozyme (T‐type) from Carolina BioSystems, and *B. subtilis* lysozyme (bacterial‐type) from NZYtech. Sigma‐Aldrich provided ampicillin sodium salt, hydroxyethyl piperazine ethane sulfonic acid (HEPES), imidazole, thrombin, ammonium nitrate (NH_4_NO_3_), formaldehyde, crystal violet, sodium chloride (NaCl), phosphate‐buffered saline (PBS), 4‐methylumbelliferone, cytochrome *c*, and LB broth powder. Solarbio supplied assay kits for ATP, NAD^+^/NADH, pyruvate, as well as defibrinated horse blood. Thermo Fisher contributed methanol, glutaraldehyde, NP40, urea, protease inhibitor, TMT‐16^plex^, C18 column, propidium iodide (PI), Triton X‐100, ethanol, SYBR gold dye, SYBR master mix, DMEM/F12, fetal bovine serum (FBS), BCA assay kit, ROS kit, IL‐6 kit, CCK‐8 assay kit, H&E staining kit, TRIzol, a microplate reader, a bacterial incubator, and a high‐throughput screening robot. GE Healthcare was the source of the fast performance liquid chromatograph (FPLC), ITC 2009, PCR machine, Superdex 75 column, HiTrap SP HP cation exchange column, dialysis membrane, and centrifugal filters (Ultra‐15 [10 and 30 kDa]). TsingKe Biotech supplied the RNeasy minelute kit and goldenstarRT cDNA synthesis mix kit. Beckman provided the ultra sonicator and centrifuges. Millipore delivered deionized water (18.2 MΩ·cm) and 0.45/0.22 μm filters. Unless otherwise noted, all other chemicals and compounds were purchased from MedChemExpress.

### Synthesis and digestion of the cell wall

According to the reported method^58^, overnight cultured MRSA was inoculated into LB medium and cultured at 37°C with shaking at 250 rpm until its OD_600_ reached ~0.6. Then, mid‐log‐phase MRSA was treated with 1.0 µg/ml cefazolin, 0.5 μg/ml cefdinir, 0.5 mg/ml lysozyme alone, or a combination of 0.5 μg/ml cefdinir and 0.5 mg/ml lysozyme. Untreated bacteria were divided into two groups, that is, a control group and a no‐HADA group. All samples were further treated with 10 µM HADA, except for those in the no‐HADA group, in which HADA was replaced with PBS. After 2‐ or 4‐h incubation, all bacteria were collected by centrifugation at 3220*g* for 10 min and then washed with cold PBS for 3 times to remove redundant HADA. The bacterial suspensions were then normalized based on OD_600_, and 100 μl of each suspension was transferred to a well of a 96‐well plate. Fluorescence intensity was measured using an M200 microplate reader with excitation at 385 nm and emission at 461 nm.

To evaluate the cell wall digestion rate, a similar bacterial treatment was performed. Subsequently, the bacteria were sonicated to disrupt the cells, and the cell wall fragments were collected by centrifugation at 3220*g* for 10 min. The cell wall pellets from different treatment groups were resuspended and normalized to an equivalent concentration in analysis buffer (50 mM NaCl, 10 mM Tris‐HCl, pH 8.0); 100 μl aliquots of all cell wall suspensions were digested with 0.5 mg/ml lysozyme at 37°C. The fluorescence intensity at the start of digestion (0 h) was defined as 100%. After 2 h of incubation, the samples were centrifuged at 3220*g* for 10 min to separate the digested fragments (supernatant) from the undigested cell wall material (cell wall pellet). The fluorescence intensity of the supernatant was measured (*λ*_ex_ = 385 nm and *λ*_em_ = 461 nm). The undigested pellet was resuspended in 100 μl of analysis buffer, and its fluorescence intensity was also recorded. The ratio (%) of the fluorescence intensity in the supernatant (representing the digested cell wall) to the initial intensity was calculated. Similarly, the ratio (%) of the fluorescence intensity in the resuspended pellet (undigested cell wall) to the initial intensity was also calculated. This process of sampling, centrifugation, and measurement was repeated at 4, 8, and 16 h. All assays were performed in triplicate, and results were expressed as mean ± standard deviation (SD).

### Proteomic assay

Exponential‐phase MRSA cells were treated with lysozyme (0.5 mg/ml), cefdinir (0.5 µg/ml), or their combination at 37°C, following a previously reported protocol^5^. A separate group received mezlocillin at 1.0 µg/ml under the same conditions. Untreated cells served as the control. After 1 h of incubation, all bacterial cells were harvested. Total protein extraction was carried out by multiple rounds of freeze–thaw in liquid nitrogen followed by sonication in a lysis buffer composed of 25 mM HEPES‐Na, 150 mM NaCl, 0.1% NP40, 4 M urea, and 1× protease inhibitor. Protein concentrations were determined using a BCA kit (Thermo Fisher), and then each sample was digested with trypsin at a 1:5 enzyme‐to‐substrate ratio overnight. The resulting peptides were labeled with TMT‐16^plex^ prior to analysis on an Orbitrap Exploris™ 480 mass spectrometer (Thermo Fisher) coupled with an UltiMate 3000 UPLC System (Thermo Fisher) equipped with a C18 analytical column. Mobile phase A contained 0.1% FA in water, and mobile phase B consisted of 0.1% FA in 100% ACN. Peptide separation was performed over 120 min at a flow rate of 300 nl/min using the following gradient: B was increased to 6% at 12 min, 20% at 82 min, 30% at 92 min, 90% at 100 min, and held at 90% for 5 min. Mass spectra were acquired in data‐dependent acquisition (DDA) mode with high‐energy collisional dissociation (HCD) fragmentation operated in TopN mode. MS1 resolution was set to 60,000, and MS2 resolution to 15,000, with a maximum injection time of 30 ms. All raw spectra were searched against the UniProt *S. aureus* database (20,330 entries, accessed 09/2019) using MaxQuant version 1.5.8.2. Search parameters included: precursor mass tolerance of 10 ppm; fragment ion tolerance ± 0.1 Da; carbamidomethylation of cysteine as a fixed modification; oxidation of methionine and protein N‑terminal acetylation as variable modifications. Trypsin was specified as the enzyme, allowing up to two missed cleavages. The false discovery rate for both peptide‐spectrum matches and proteins was set at 1%. A maximum of three modifications per peptide was permitted. DEPs were defined as those with a *p*‐value ≤ 0.05 and a fold change (FC) ≥ 1.5 (log_2_FC ≥ 0.58 or log_2_FC ≤ −0.58). Comparisons between treatment groups were performed using the cuff‐diff program.

PRM analysis was conducted following a similar protocol with slight modifications^5^. Label‑free peptides from overnight digestion were loaded onto the same Orbitrap Exploris™ 480 mass spectrometer. PRM methods were directly derived from the DDA data; the latter were imported into Skyline to select target peptides of interest. For each selected peptide, information on precursor *m/z*, charge state, and retention time window was exported from Skyline to Xcalibur software to build the PRM method. Key MS1 parameters were: 60,000 resolution, and 2.0 × 10^5^ AGC target, 50 ms maximum injection time. PRM scans were performed at 30,000 resolution, 1 × 10^5^ AGC target, 54 ms maximum injection time, and 0.7 *m/z* isolation window with peptides in 5 min retention time window. Acquired PRM data were re‑imported into Skyline for validation of the proteomic findings. To ensure reliable identification and quantification, idotp and dotp values were required to be >0.60. Peptide abundance was calculated by summing the intensities of the top three product ions.

### Cytochrome *c* binding assay

According to the reported method^34^, ^59^, overnight cultured MRSA was inoculated into LB medium at a ratio of 1:1000. After 3.5 h incubation at 37°C, mid‐log‐phase MRSA (OD_600_ ~ 0.6) was exposed to cefazolin (0.5 μg/ml), cefdinir (0.5 μg/ml), lysozyme (0.5 mg/ml) alone, or their combination (0.5 μg/ml cefdinir and 0.5 mg/ml lysozyme). Those without any treatment served as the control group. After 1 h incubation at 37°C, all bacteria were washed twice in deionized water and suspended in the same solution until the final OD_600_ reached 2.4. The suspension was then incubated with 0.5 mg/ml of cytochrome *c*. After 10 min incubation at room temperature, the supernatant was collected by centrifugation at 12000*g* for 3 min. Deionized water containing 0.5 mg/ml of cytochrome *c* served as the positive control; 200 μl of supernatant from each group was added to each well to analyze the amount of unbound cytochrome *c* by measuring absorbance at 530 nm. Meanwhile, a similar protocol was performed in the negative group, but lacking 0.5 mg/ml of cytochrome *c*. Notably, the lower intensity indicated stronger binding and thus more negative charges in the cell wall. All assays were performed in triplicate, and results were expressed as mean ± SD.

### The SEM imaging analysis

According to the standard method^60^, an MRSA single colony was picked up and cultured in LB medium. After 24 h incubation at 37°C, the bacterial suspension was inoculated into LB medium containing cefdinir (0.5 μg/ml, 5 h), lysozyme (0.5 mg/ml, 5 h) alone, or their combination (5 h). An untreated MRSA suspension served as the control group. After 5‐h incubation in a shaking at 250 rpm at 37°C, MRSA in log phase was collected by centrifugation at 3220*g* for 10 min and fixed with 2.5% glutaraldehyde for 2 h at 4°C prior to being washed and resuspended in PBS. After that, bacterial suspensions were dehydrated by using increasing concentrations of ethanol in water (10%, 30%, 50%, 70%, 80%, 90%, and 100%) for 10 min in each step. Then, 5 μl of bacterial suspension at 1.0 × 10^9^ CFU/ml was incubated on polyethylenimine‐coated coverslips (22 mm × 22 mm), and the coverslips underwent drying in a Balzers critical point dryer (Balzers) prior to mounting on 25 mm aluminum stubs with double‐sided carbon tabs. The edges of each coverslip were treated with silver liquid, allowed to dry, and then gold‐coated in an Edwards S150B sputter coater (Edwards High Vacuum, Crawley) for 60 s. Imaging was performed with a TESCAN VEGA3 scanning electron microscope (Brno) at a voltage of 20 kV. Following image acquisition, a minimum of 20 cells were randomly selected from multiple fields of view for each treatment group to ensure statistical significance. Representative images under the same magnification from each group were selected for presentation. The degree of cell surface deformation and rupture was assessed qualitatively and categorized based on the severity of morphological alterations.

### Cell‐based infection model and cytotoxicity analysis

Both ARPE‐19 and 293 T cells were first cultured in Gibco Dulbecco's Modified Eagle Medium: Nutrient Mixture F‐12 (DMEM/F12) supplemented with 10% fetal bovine serum (FBS) and grown at 37°C in 5% CO_2_‐humidified atmosphere for 7 days. Then, about 1.0 × 10^4^ collected cells were seeded into each well of 96‐well plates and incubated overnight to ensure their confluency. Logarithmic cultures of MRSA were washed three times with PBS and resuspended in DMEM/F12 containing 10% FBS to obtain bacterial suspensions with an initial density of about 5.0 × 10^7^ CFU/ml. Next, 10 μl of bacterial suspension was added to each well and incubated for another 3 h (MOI of 50). Next, infected cells were washed vigorously with PBS six times and replenished with culture medium to remove unbound bacteria. Herein, cell‐associated bacteria were defined as bacteria that attach to, penetrate, or transcytose in cells. Next, these cells were exposed to either lysozyme, cefdinir, or their combination overnight under identical cell culture conditions (100 μl per well). Uninfected cells in the absence of drugs served as the positive control group. Infected cells without any drug treatment served as the negative control group. After 24 h of incubation, CCK‐8 assays were performed to analyze cell survival under different treatments. Specifically, 10 μl of CCK‐8 solution per well was added, and these cells were incubated for another 2 h at 37°C for chromogen development. The absorbance was measured at 450 nm, with 630 nm as the reference wavelength. Herein, survival ratio = (Abs_sample_–Abs_negative control_)/(Abs_positive control_–Abs_negative control_) × 100%. The bacterial loads were examined by lysing cells with 1% Triton X‐100 in PBS and serially diluting the resulting lysates to enumerate bacterial colonies on agar plates. To analyze the cytotoxicity of lysozyme and cefdinir, a similar protocol was followed^61^, although neither ARPE‐19 nor 293 T cells were infected with any bacteria. All assays were performed in triplicate, and results were expressed as mean ± SD.

### Rat skin infection model

According to the reported method^20^, 25 Sprague–Dawley rats (6–7 weeks old) were divided into five groups (5 rats per group). Five rats served as the uninfected group, while the others were infected with about 5 × 10^11^ CFU of mid‐log phase MRSA. Specifically, all rats were first pre‐depilated by using 10% Na_2_S to get an uncovered back area of 4 cm × 4 cm prior to 24 h for the anesthetization of rats. Then, the skin of anesthetized rats was removed down to the fascial region under sterile conditions, followed by spraying 1.25 ml of bacterial suspension onto the wound (3 cm × 3 cm). To collect these bacterial suspensions, mid‐log phase MRSA was collected by centrifugation at 4500*g* for 15 min, washed with PBS for 3 times, and resuspended in cold PBS to get a bacterial suspension with an initial density of about 4.0 × 10^11^ CFU/ml according to the pre‐experiment. Normal saline was used to replace the bacterial suspension in the uninfected group. After 1 h of infection, the wounds of infected rats were covered with sterile medical gauze. All sterile medical gauzes were 0.2 g/cm^2^, which were further covered by two layers of sterile gauze and fixed by sterile tape. After that, rats were monitored until they were fully awake, followed by their return to their cages. After 1 dpi, these sterile medical gauzes were replaced with new sterile medical gauzes. Specifically, rats in lysozyme only and combination groups were treated with new sterile medical gauze soaked in lysozyme (70 mg/kg, ~14 mg). However, rats in the other groups were given the same type of sterile medical gauzes, namely, gauzes soaked with normal saline. Meanwhile, cefdinir was prepared in 0.5% methylcellulose and was further administered orally (12.5 mg/kg) to rats in cefdinir‐only and combination groups. All treatments were repeated at 3 dpi. At 7 dpi, all rats were sacrificed to collect their wounds. These collected tissues were fixed in 10% formalin for at least 24 h and embedded in paraffin. About 4‐μm sections were analyzed using H&E staining. The remaining wounds were placed into 1 ml sterile PBS on ice, and then homogenized to detect the levels of ROS and IL‐6. The rest of the homogenates was immediately stored in a sterile tube at −80°C for further analysis.

### Statistical analysis

All assays were performed in triplicate, and results were expressed as mean ± SD unless otherwise stated. Statistical comparisons between two groups were performed using an unpaired two‐tailed Student's *t*‐test. All tests of significance were based on *p < 0.05, **p < 0.01, and ***p < 0.001.

## AUTHOR CONTRIBUTIONS

**Qi Zhang:** Conceptualization; methodology; project administration; validation; visualization; writing—original draft preparation, review and editing. **Yang Yang:** Methodology; visualization. **Shuqi Li:** Methodology. **Zhao Liu:** Writing—review and editing. **Chun‐Kit Lee:** Writing—review and editing. **Chi‐Bun Ko:** Writing—review and editing. **Qian Zhao:** Funding acquisition; writing—review and editing.

## ETHICS STATEMENT

The protocol to perform animal study has been reviewed and approved by and performed in accordance with the guidelines approved by the Committee on the Use of Live Animals in Teaching and Research (CULATR) (Ref No. ARSA‐22127‐OTH‐ABCT), The Hong Kong Polytechnic University, China.

## CONFLICT OF INTERESTS

Qian Zhao and Qi Zhang have filed a patent application (US application No. 18/888,874) related to the work presented in this manuscript. All other authors declare no conflict of interest.

## Supporting information

Updated SI.

Table S1.

Table S2.

Table S3.

## Data Availability

All data generated or analyzed during this study are included in the paper or the supplementary materials. For additional materials, please contact the corresponding author.
