# Supplementary material for: Cefdinir reprograms Gram‐positive bacteria to synergize with lysozyme against superbugs
Source: mLife. 2026 Jun 12;5(3):355–68. doi: 10.1002/mlf2.70080 (PMC13327612; doi:10.1002/mlf2.70080)
Supplement: Supplementary file 1 — Updated SI. [file MLF2-5-355-s002.docx]

***Supplementary information* for**

**Cefdinir reprograms Gram-positive bacteria to synergize with lysozyme against superbugs**

Qi Zhang^1,2^, Yang Yang^1,2^, Shuqi Li^1^, Zhao Liu^1,2^, Chun-Kit Lee^1^, Chi-Bun Ko^1^, Qian Zhao^1,2#^

1 State Key Laboratory of Chemical Biology and Drug Discovery, Department of Applied Biology and Chemical Technology, The Hong Kong Polytechnic University, Hong Kong, China

2 Centre for Eye and Vision Research (CEVR), Hong Kong Science Park, Hong Kong, China

^#^Corresponding author: Qian Zhao; q.zhao@polyu.edu.hk

**Materials and Methods**

1. **High-throughput screening**

According to standard compounds-screening method[1], 4,934 compounds (6 sub-classes) from our drug library at the fixed concentration of 2.5 µM were screened against MRSA or MRSE in combination with human lysozyme. Specifically, bacterial suspensions at a concentration of 1×10^6^ CFU/ml in the 96-well microliter plate were incubated with these compounds, in which each well contained 50 µl of LB medium with or without 1 mg/ml lysozyme. LB medium in the absence of bacteria served as background group. Then, the real-time growth curves were monitored per hour and for 22 hours. Experiments were performed with three biological replicates. By analyzing the absorbance at 600 nm (OD_600_) of bacterial culture at the time point of 24-hour, combination inhibition ratio (%) was calculated according to (OD_compound alone_−OD_combination_)/(OD_compound alone_−OD_background_) ×100%. Synergy was defined as inhibition ratio of ≥ 90%. Each test was performed in triplicate.

1. **Checkerboard broth micro-dilution assays**

According to standard two-fold dilution method[2], cefdinir at different concentration was added into 180 µl fresh LB medium (or trypticase soy broth medium with 5% defibrinated horse blood) containing different concentration of human lysozyme in 96-wells plate. After well mixed, about 20 µl of 1.0×10^7^ CFU/ml logarithmic cultures of MRSA, MRSE, *Streptococcus pyogenes*, *Streptococcus mutans*, *Enterococcus faecium*, *Enterococcus faecalis*, *Corynebacterium pseudodiphtheriticum*, *Klebsiella pneumoniae*, *Pseudomonas aeruginosa* and *Escherichia coli* were added into each well of the plate and co-incubated overnight. Bacterial suspensions without any drug treatments served as growth controls, and LB medium without bacteria served as the background group. The inhibition ratio was calculated as (OD_control_−OD_sample_)/(OD_control_−OD_background_)×100%. Besides, bacterial growth inhibition was also monitored by serial dilution in LB-agar plate. The CFU was counted by performing 10-fold serial dilution in PBS and then spotting 10 µl of dilutions in LB-agar plate. The MIC was determined as the lowest concentration of a drug that could inhibit the 90% growth of microorganism by both visual CFU and OD_600_ using a microtiter plate reader. Fractional inhibitory concentration (FIC) and FICI values were determined by standard method[1]. Briefly, FICI= MIC_AB_/MIC_A_+MIC_BA_/MIC_B_ =FIC_A_+FIC_B_. MIC_A_ was the MIC of compound A alone; MIC_AB_ was the MIC of compound A in combination with compound B; MIC_B_ was the MIC of compound B alone; MIC_BA_ was the MIC of compound B in combination with compound A; FIC_A_ was the FIC of compound A; and FIC_B_ was the FIC of compound B. Herein, synergy, part synergy and non-synergy were defined as an FICI of ≤0.5, 0.5<FICI<1 and FICI≥1, respectively. Each test was performed in triplicate.

1. **Time-killing curves**

According to standard method[2], overnight-cultured MRSA was diluted and aliquoted into new sterile 50 ml tubes at 1:1000 ratio. Then, the bacterial suspension was cultured in LB medium containing 0.5 or 2.0 μg/ml cefdinir, 0.5 mg/ml lysozyme or their combination at 37 °C with shaking at 250 rpm. Bacterial suspension without any drugs served as control group. To measure their kinetic time-killing curves, 20 µl of each culture was extracted at time intervals of 0-, 2-, 4-, 6-, 9- and 16- hour for measurement of the OD_600_ and CFUs. 10 µl of each dilution was spotted on the LB-agar plates and incubated at 37 °C for 24 hours prior to enumeration. All assays were performed in triplicate.

1. **Mutation frequency and prevention concentration analysis**

According to previously described method[1], MRSA culture in log phase were collected and concentrated into bacterial suspension of about 1.0×10^10^ CFU/ml in PBS buffer. Then, 100 µl of diluted MRSA suspension was evenly applied onto agar plates with gradient cefdinir concentrations (including 1, 2, 4 and 8 μg/ml) in the presence of lysozyme (including 0.5, 1, 2 and 4 mg/ml). Diluted bacterial culture on agar plates with cefdinir alone served as control group. Initial diluted MRSA suspension was also cultured on agar plate without any additions to figure out accurate bacterial concentrations. After 48 hours incubation at 37°C, colony counts on agar plates were figured out. Herein, the bacterial mutation frequency was calculated as colony countstreated group/colony countsinitial supplied group. Besides, the minimal concentrations of cefdinir combined with lysozyme in plates without any colony were recorded as mutation prevention concentrations (MPC) in combination therapy. Corresponding quotients between MPC and cefdinir itself MIC in control group were figured as “mutation prevention index” (MPI). All assays were performed in triplicate.

1. **Biofilm assays**

According to the report method[1], biofilm inhibition was assessed by using an adherence assay on 96-well plate. Briefly, to evaluate the combination effect on pre-biofilm, bacterial suspensions (5×10^6^ CFU/ml) were exposed to different concentrations of cefdinir in LB medium in the absence and presence of 0.5 mg/ml lysozyme. LB medium with or without bacteria served as control or background group, respectively. After overnight incubation at 37 °C, bacterial suspensions were removed and these wells were gently washed twice with sterile PBS to remove exclusively non-adherent bacteria. The adherent biofilms were fixed by using 95 % methanol at 60 °C for 15 minutes. Then, biofilms were stained with 1 % crystal violet (100 μl per well) at 37 °C for 15 minutes, followed by gently washing with PBS to remove redundant crystal violet. Finally, the dye was solubilized with 150 μl of 95 % ethanol per well at 37 °C for 30 minutes. Optical density of solution in each well was measured at 590 nm using a micro-plate reader. Strong biofilm, weak biofilm and no biofilm were defined as “OD_sample_≥ 2OD_background_”, “OD_background_<OD_sample_<2OD_background_” and “OD_sample_≤OD_background_”, respectively. To evaluate the combination effect on post-biofilm, adherent bacteria were pre-separated from 24-hour bacterial medium without any drugs. Then, sterile LB medium containing different concentrations of cefdinir in the absence and presence of lysozyme were added into these wells for future 24-hour culture. Next, the absorbance (OD_600_) was measured to analyze the growth of the bacterial culture in the post-biofilm. All assays were performed in triplicate.

1. **Cell wall and cell membrane integrity**

To analyze the integrity of cell wall, an aliquot of each sample was diluted into the melted agarose (1.25×10^5^ CFU/ml, 0.5 ml). Next, 20 μl aliquot (5×10^3^ CFU in total) of the sample-agarose mixture was pipetted onto a precoated slide, and the sample was covered with a 22×22 mm coverslip. The slide was placed on a cold plate in the refrigerator (4°C) for 5 minutes to allow the agarose to produce microgel with the trapped intact cells inside. The coverslip was removed gently, and the slide was immediately immersed horizontally in the lysing solution (20 mM HEPES-Na, 0.5 % NP40 and 0.5 % Triton-X100) for 5 minutes at 37°C. The slide was washed horizontally in a tray with abundant distilled water for 3 minutes, dehydrated by incubating horizontally in cold ethanol with increasing concentrations (70%, 90% and 100%) for 3 minutes each time. Then, dried slide was stained with 25 μl of SYBR-Gold (Thermo fisher, China) diluted 1:400 in TBE buffer for 2 minutes in the darkness. After a brief wash in PBS buffer, a 24×60 mm coverslip was added, and the slides were visualized under fluorescence microscopy.

To analyze the integrity of cell membrane, mid-log-phase MRSA under different pre-treatments were incubated with propidium iodide (PI) at final concentration of 0.5 μM. After incubation for 30 minutes in the darkness, the fluorescence intensity (λ_ex_=535 nm, λ_em_=615 nm) was determined. Those treated with 0.5% Triton-X100 or 1×PBS served as positive or negative control group. All assays were performed in triplicate and results were expressed with fluorescence intensities as mean ± SD.

1. **RNA isolation and RT-qPCR**

According to the reported method[3, 4], the total RNA was extracted from MRSA under different treatments for 1 hour by using TRIzol kit and RNeasy minelute kit. Reverse transcription was conducted with 1 μg of total RNA using the goldenstarRT cDNA synthesis mix kit. Then, the real-time quantitative polymerase chain reaction (RT-qPCR) was performed using the SYBR master mix. Relative levels of genes expression were quantified using the comparative C_T_ method, where the C_T_ values were normalized with the housekeeping gene *rpoB* for comparison[5]. All assays were performed in triplicate and results were expressed as mean ± SD.

1. **Purification of G-type lysozyme**

According to a referable method with refinement[6], goose egg white (from *Anser cygnoides* egg) was firstly separated, diluted with two volumes of 50 mM PBS (pH 7.0) and stirred for 30 minutes at 4 ^o^C, followed by centrifugation at 12,000 *g* at 4 °C for 15 minutes to collect the soluble supernatant. Next, the crude extract was further treated with isoelectric precipitation at pH 4.0, 6.0, and 7.0. At each step of pH treatment, the solution was adjusted to the desired pH by using 1 M HCl or NaOH for next incubation at 4 ^o^C for 1 hour. After that, the crude extract would be further purified by centrifuging at 12,000 *g* at 4 °C for 30 minutes and filtering under 0.45 µm filter to remove insoluble protein precipitation. Next, all soluble supernatant was applied to a 5 ml HiTrap SP-FF cation exchange resin column (GE Healthcare) pre-washed by five column volumes of 50 mM PBS (pH 7.0) at a rate of 2 ml/min. Then, column was washed with the same buffer and the targeted lysozyme was eluted with a linear gradient of NaCl concentration from 100 mM to 500 mM in the same buffer at the flow rate of 1.5 ml/min. Next, the purified lysozyme was pooled and uploaded onto Superdex 75 column (GE Healthcare) equilibrated with running buffer (10 mM NH_4_NO_3_, pH=7.0). These target lysozyme fractions were collected and then concentrated to 80 mg/ml for further analysis using SpeedVac rotary evaporator at 4 °C for 5 hours, followed by aliquoting for long-term storage at −80 °C.

1. **Acetylation evaluation**

To analyze the potential change in enzymes involving in acetylation of cell wall in different groups, mid-log-phase MRSA with OD_600_ of 0.4 to 0.6 was treated with 0.5 mg/ml lysozyme, 0.5 μg/ml cefdinir or their combination. Those without any treatment served as control group. After 1 hour incubation at 37 °C, equal bacteria (1 ml bacteria were collected when the OD_600_ was 1) were collected and washed by cold PBS for 3 times, followed by ultra-sonication to collect the supernatant. According to described method[7] and previous pre-assay, 1.5 μg of cell supernatant was incubated with 0.5 mM of 4-Methylumbelliferyl acetate (4-MU-Ac). After 3 hours incubation at 37 °C, 200 μl of the mixture in each group was added into each well to analyze the fluorescence intensity (λ_ex_=372 nm, λ_em_=450 nm). Those cell supernatants from control group were used in pre-assay to figure out the linear range. All assays were performed in triplicate and results were expressed as mean ± SD.

1. **ATP, NAD^+^/NADH and pyruvate levels**

Mid-log-phase MRSA under different drug treatments (1 hour at 37 °C) was washed by cold PBS for 3 times. Next, to evaluate the pyruvate level, all samples were re-suspended in cold extracting buffer, followed by sonication to collect these supernatants. Then, 75 µl of each supernatant was incubated 25 µl of reaction I, followed by continuous incubation with 125 µl of reaction II. By recording the absorbance at 520 nm, the pyruvate level was figured out according to the standard formula. To evaluate the intracellular ATP, NAD^+^/NADH levels, similar protocol was performed to collect supernatants. Then, 20 µl of each supernatant was added white 96-well plate pre-reactivated by 100 µl of ATP test solution, followed by luminometer measurement. To analyze the intracellular NAD^+^ and NADH levels, each lysis supernatant was divided into two tubes. One tube was heated at 60 °C for 30 minutes in PCR machine to decompose NAD^+^ if had. Then, this tube was centrifuged at 18, 000 *g* at 4 °C for 20 minutes to collect the supernatant. After that, 20 µl of supernatant from heated or unheated group was added white 96-well plate pre-reactivated by 90 µl/well of alcohol dehydrogenase solution for 10 minutes incubation at 37 °C in the prevention of light. Next, 10 µl of staining solution was added to react with product in previous reaction. After 30 minutes at 37 °C in the prevention of light, orange formazan was observed and then the absorbance at 450 nm was recorded. Intensities of those with and without heat treatment indicated the levels of NADH+NAD^+^ and NADH itself, respectively. Finally, the intracellular levels of NAD^+^, NADH and their ratio were figured out. All kits were ordered from Beyotime, China. Assays were performed in triplicate and results were expressed as mean ± SD.


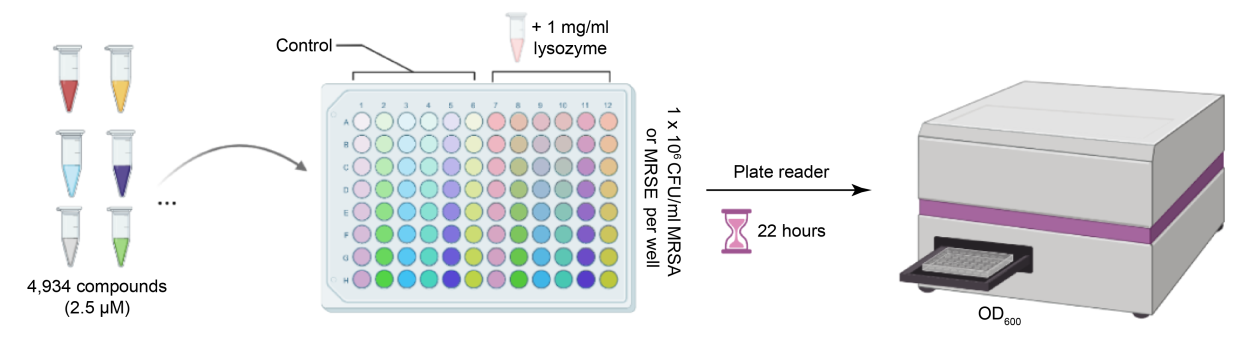


**Figure S1.** The schematic for screening novel adjuvants of widely used lysozyme from a drug library containing 4,934 compounds. All compounds (6 sub-classes) at the fixed concentration of 2.5 μM were screened against MRSA or MRSE in the presence of 1 mg/ml lysozyme. Bacteria treated with these screened compounds in the absence of lysozyme served as relative background groups. Real-time bacterial growth curves were monitored for 22 hours. The inhibition ratio (%) was calculated as (OD_compound alone_−OD_combination_)/(OD_compound alone_−OD_background_)×100%. A synergistic effect was defined as the inhibition ratio of ≥ 90%.


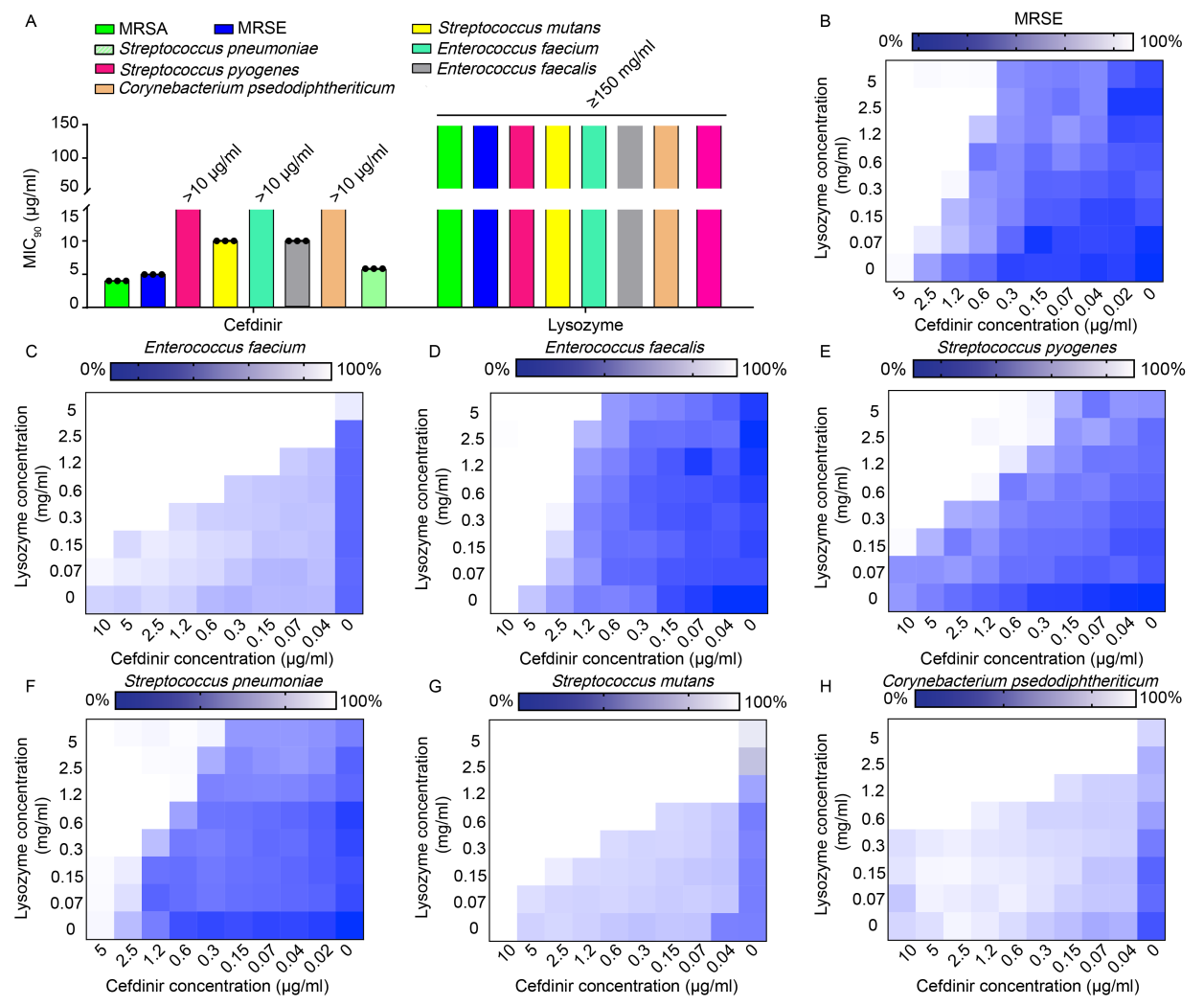


**Figure S2**. Cefdinir demonstrated a synergistic effect with lysozyme against broad-spectrum multidrug resistant Gram-positive bacteria. (A) The MIC_90_ of lysozyme and cefdinir against eight multidrug resistant Gram-positive pathogens. (B-H) Representative heat maps illustrating the effects of combination therapies on these multidrug resistant Gram-positive pathogens. The inhibition ratios of bacterial growth are represented by their upper columns.


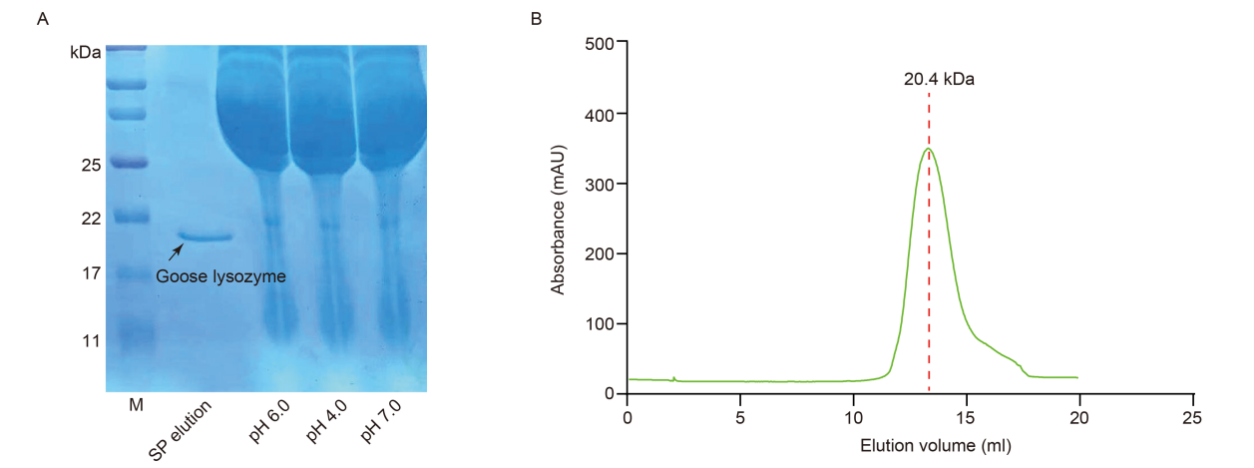


**Figure S3.** G-type lysozyme was successfully purified from eggs of Chinese geese (*Anser cygnoides*). (A) The Coomassie blue stained gel showed the increasing purity of lysozyme from goose egg white treated with isoelectric precipitation at pH 6.0, 4.0, 7.0, followed by cation-exchange chromatography (SP column). Purified lysozyme had been confirmed by using in-gel digestion mass spectrometric identification. (B) The curve of gel filtration chromatography for purifying lysozyme from the SP column elution.

**
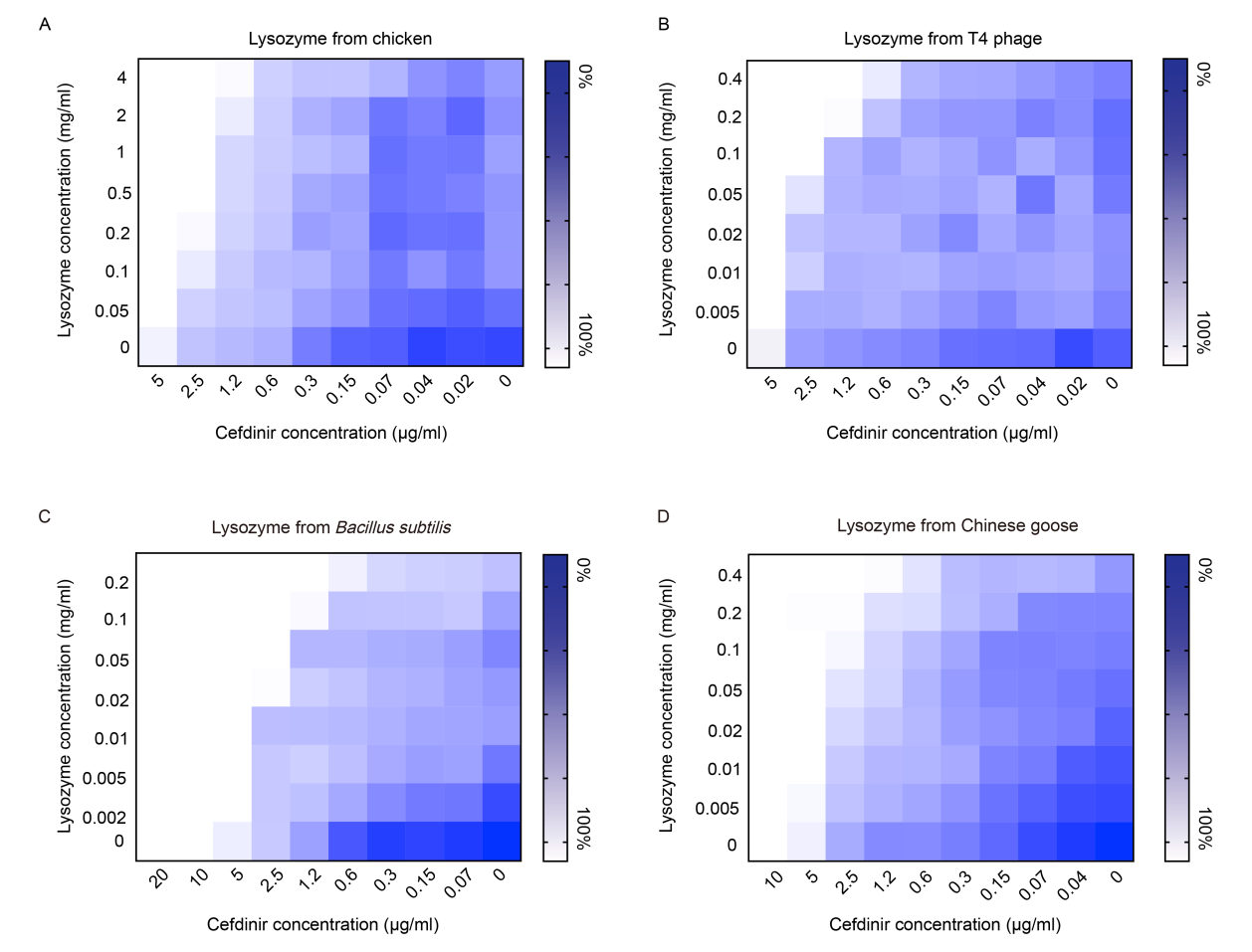
**

**Figure S4.** Representative heat maps showing (partial) synergistic effects of combination therapies. They consist of cefdinir and one type of lysozyme, *i.e.,* (A) C-type lysozyme (from chicken), (B) T4-type lysozyme (from T4 phage), (C) bacterial-type lysozyme (from *Bacillus subtilis*) and (D) G-type lysozyme (from Chinese goose). Inhibition ratios of bacterial growth were indicated by their right columns.

**
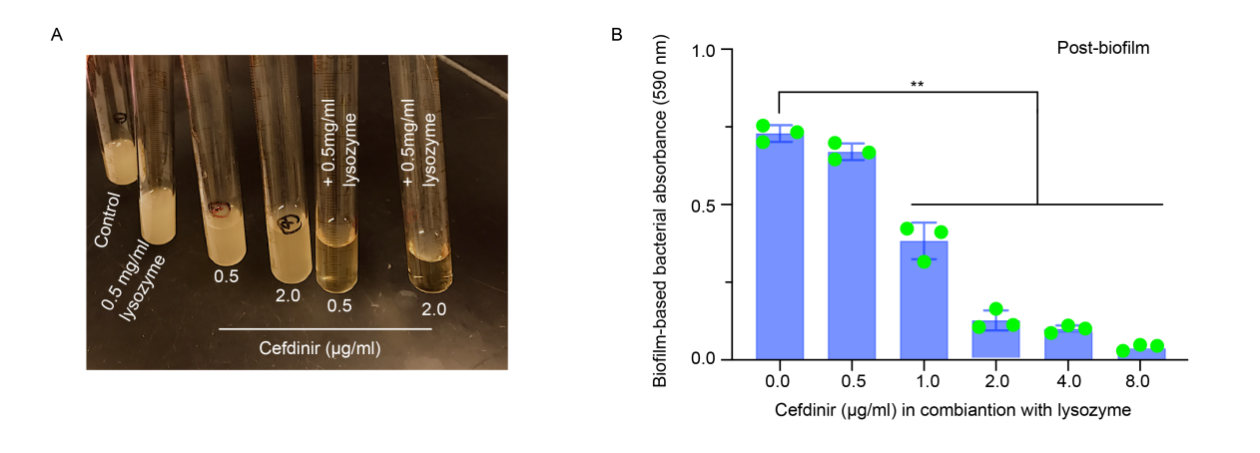
**

**Figure S5.** The bactericidal effect of combination therapy *in vitro*. (A) Representative images illustrating the time-killing effects on MRSA treated with lysozyme, cefdinir alone or their combination. (B) Biofilm-based absorbance showing the inhibitory effects of cefdinir in the presence of 0.5 mg/ml lysozyme on biofilm after its formation. ^*^*p*<0.05, ^**^*p*<0.01 and  ^***^*p*<0.001.


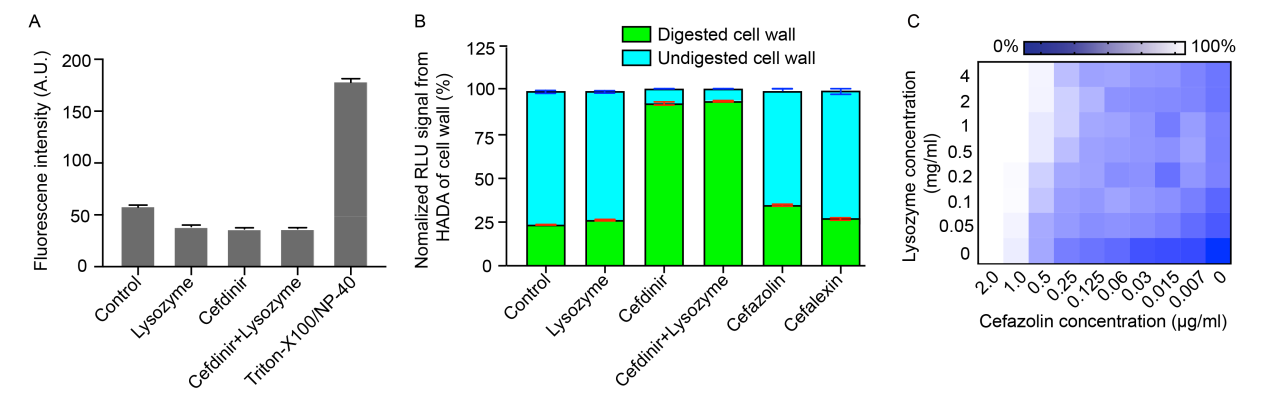


**Figure S6.** Combination therapy disrupted cell walls rather than the cell membrane. (A) Minimal damage to the cell membrane was observed under combination therapy. Herein, MRSA was exposed to 0.5 mg/ml lysozyme, 0.5 μg/ml cefdinir, and their combination for 1 hour. Triton-X100/NP-40 was used to disrupt the cell membrane in the positive control group. (B) Variations in HADA-based signal intensities suggested that the cell wall would be more susceptible to lysozyme when MRSA was treated with cefdinir. All bacteria subjected to different treatments were ultrasonicated to isolate their insoluble cell walls. Subsequently, all cell walls were further digested with 0.5 mg/ml lysozyme for 16 hours to separate two fractions (digested and undigested cell walls) via centrifugation. Bacteria treated with cefazolin or cefalexin (two randomly selected representative β-lactam antimicrobials) served as the negative control groups. (C) Representative heat maps showing the combination effect between lysozyme and cefazolin against MRSA.


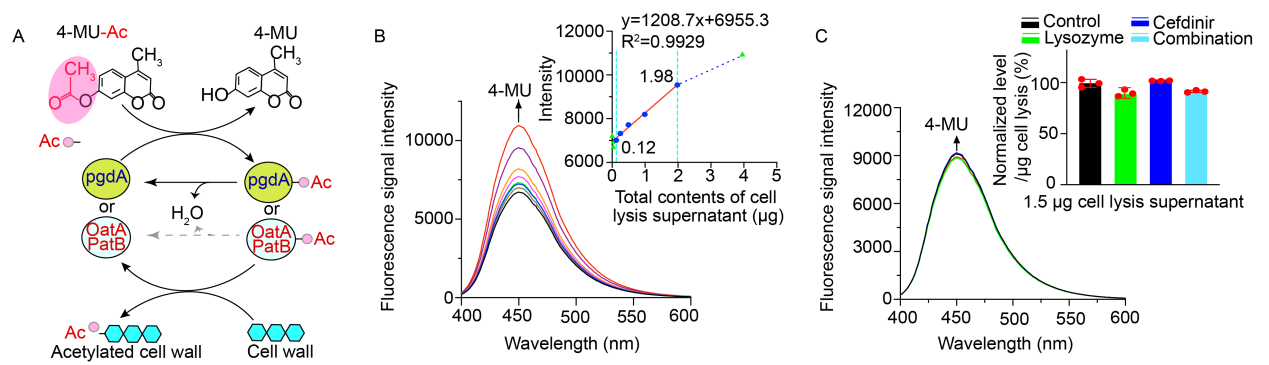


**Figure S7.** Acetylation modification analysis suggested that the effectiveness of combination therapy might be attributed to other factors. (A) A diagram illustrating the 4-MU-Ac-based reaction catalyzed by enzymes associated with the typical acetylation modification of the cell wall. Notably, compared to the dashed line, the solid line indicates the preferred substrate and reaction direction. (B) Fluorescent curve analysis of 4-MU-Ac content, indicating a linear range in absorption intensity. (C) Fluorescent spectrum of the reaction mixture containing 0.5 mM 4-MU-Ac and 1.5 μg of cell lysis supernatant from MRSA under various treatments, indicating few effects on acetylation levels under combination therapy.


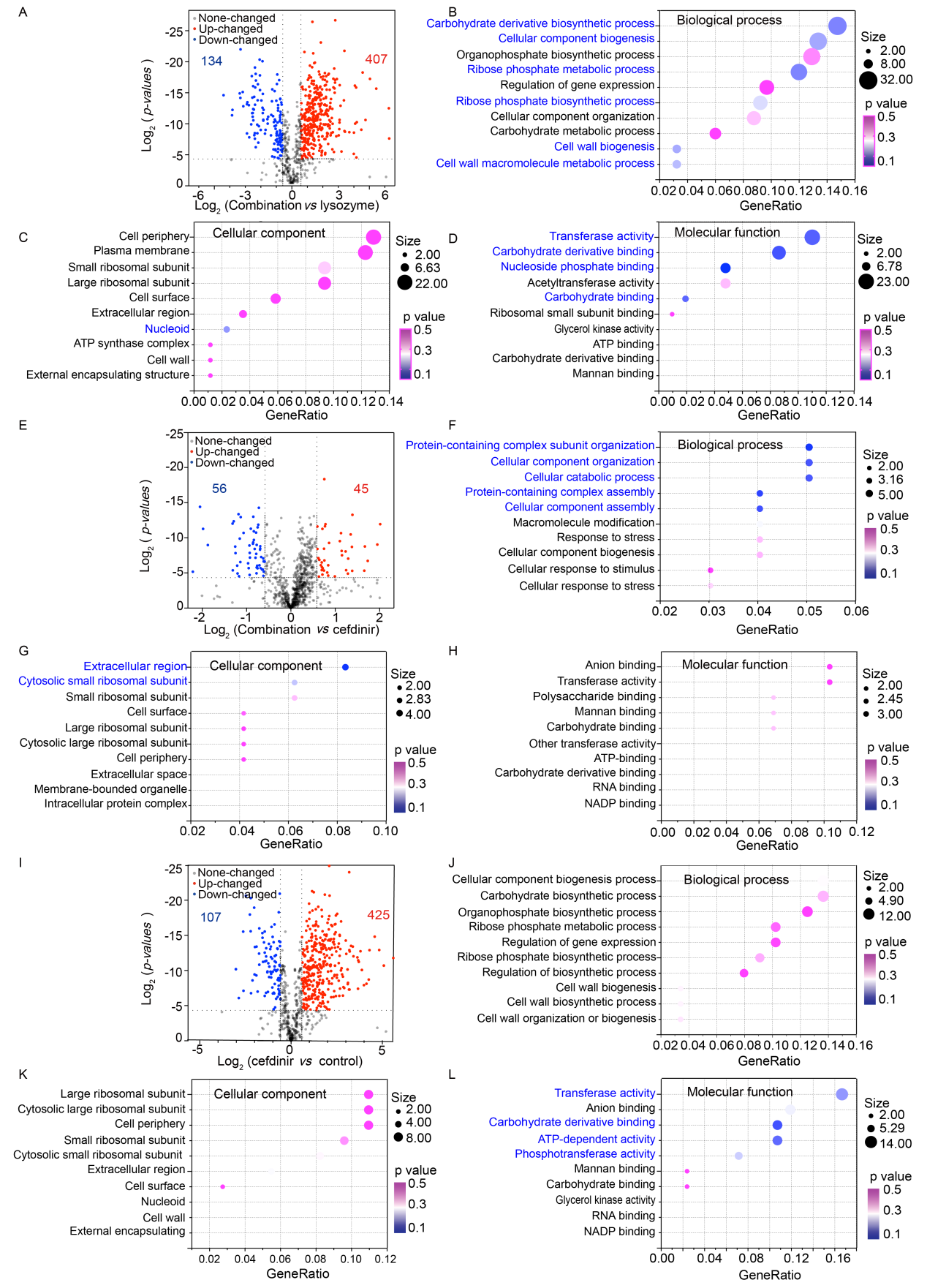


**Figure S8.** Proteomic analyses of MRSA under different treatments. (A, E, I) Volcano plots comparing protein expression between: (A) Combination vs lysozyme only treatment, (E) Combination vs cefdinir only treatment, (I) Cefdinir only vs control groups. (B-D, F-H, J-L) Corresponding GO annotation analyses for the comparisons between: (B-D) Combination vs lysozyme only treatment, (F-H) Combination vs cefdinir only treatment, (J-L) Cefdinir only vs control groups. Data information: The x- and y- axes in (A, E, I) represent changes in expression levels and corresponding statistical significance, respectively. Adjusted *p* value <0.05 (Student’s t-test) and |Fold change| >1.5 were applied as the cutoff for these DEPs.

**
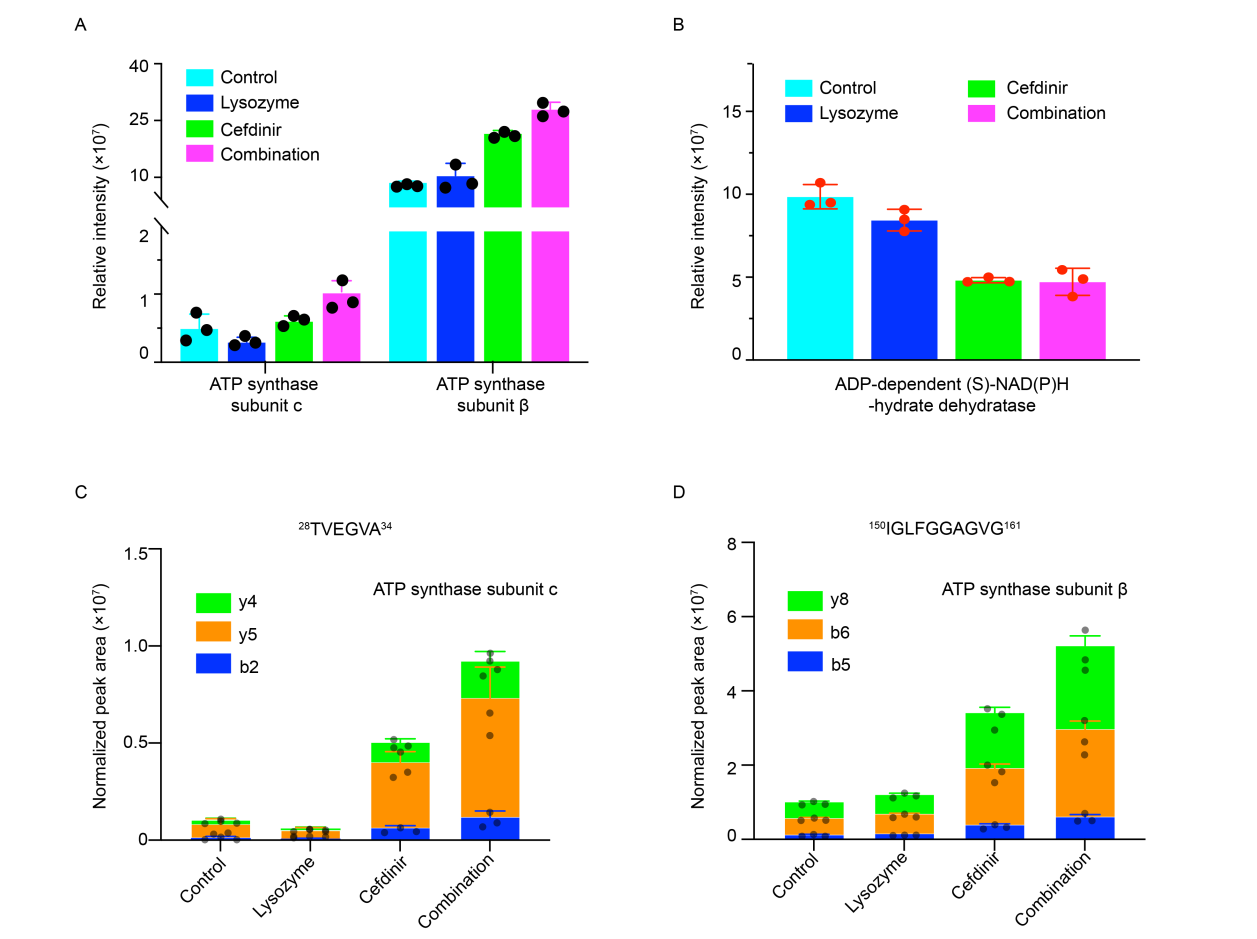
**

**Figure S9.** Proteomic analyses on MRSA under different treatments. Relative levels of (A) ATP synthase subunit c, subunit β, and (B) ADP-dependent (S)-NAD(P)H-hydrate dehydratase. (C-D) PRM-based analysis of (C) ATP synthase subunit c and (D) ATP synthase subunit β. Each inserted sequence represented the unique peptide in PRM analysis. Notably, these DEPs in these volcano plots were highly correlated with energy metabolism


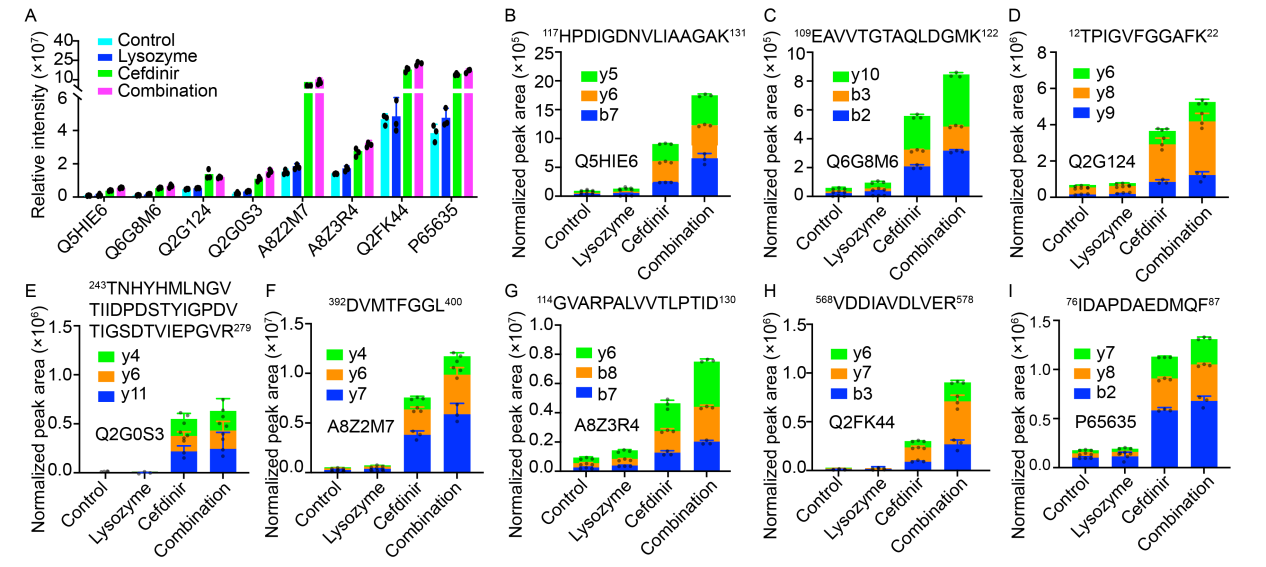


**Figure S10.** Proteomic analyses on MRSA under different treatments. (A) Fold-changes of several proteins in MRSA under different treatments. (B-I) PRM-based analyses of (B) serine acetyltransferase (UniProt ID: Q5HIE6), (C) acetyl-coenzyme A carboxylase carboxyl transferase subunit beta (UniProt ID: Q6G8M6), (D) probable acetyl-CoA acyltransferase (UniProt ID: Q2G124), (E) bifunctional protein GlmU (UniProt ID: Q2G0S3), (F) acetate kinase (UniProt ID: A8Z2M7), (G) phosphate acyltransferase (UniProt ID: A8Z3R4), (H) formate acetyltransferase (UniProt ID: Q2FK44) and (I) dihydrolipoyllysine-residue acetyltransferase component of pyruvate dehydrogenase complex (UniProt ID: P65635). The inserted sequences were these unique peptides in PRM analyses.


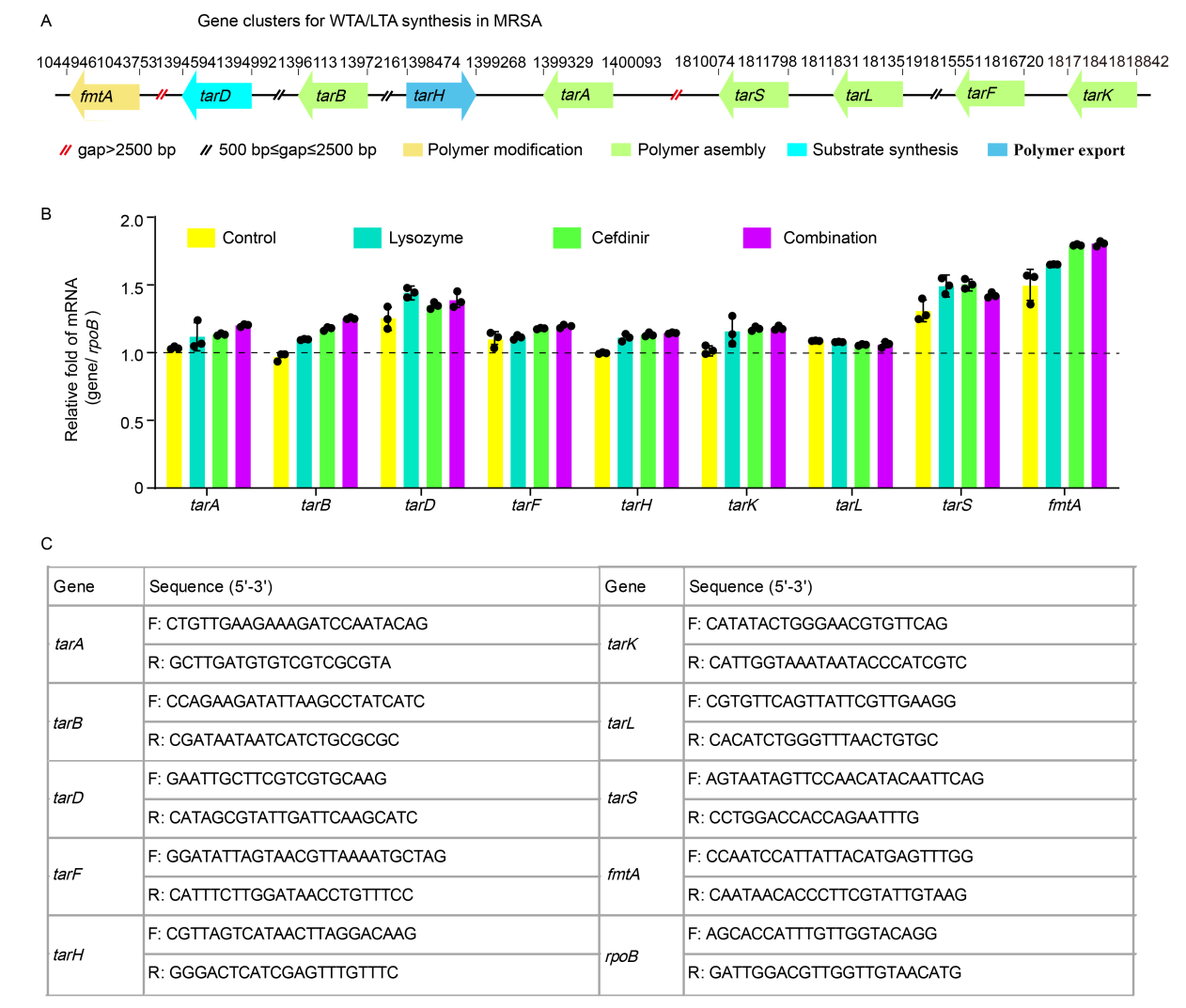


**Figure S11.** Transcriptional analysis of genes involved in bacterial WTA and LTA synthesis. (A) The location of related gene clusters in MRSA. Different colors indicated roles of encoded enzymes in WTA/LTA syntheses. The numbers flanking certain gene represent its start and end positions. (B) Relative transcription levels of genes in MRSA under various treatments. All genes were quantified using their C_T_ values and normalized to the housekeeping gene *rpoB*, which indicated minimal changes in mRNA levels of these genes regardless of the treatment conditions. (C) The primers utilized in the RT-qPCR assays.


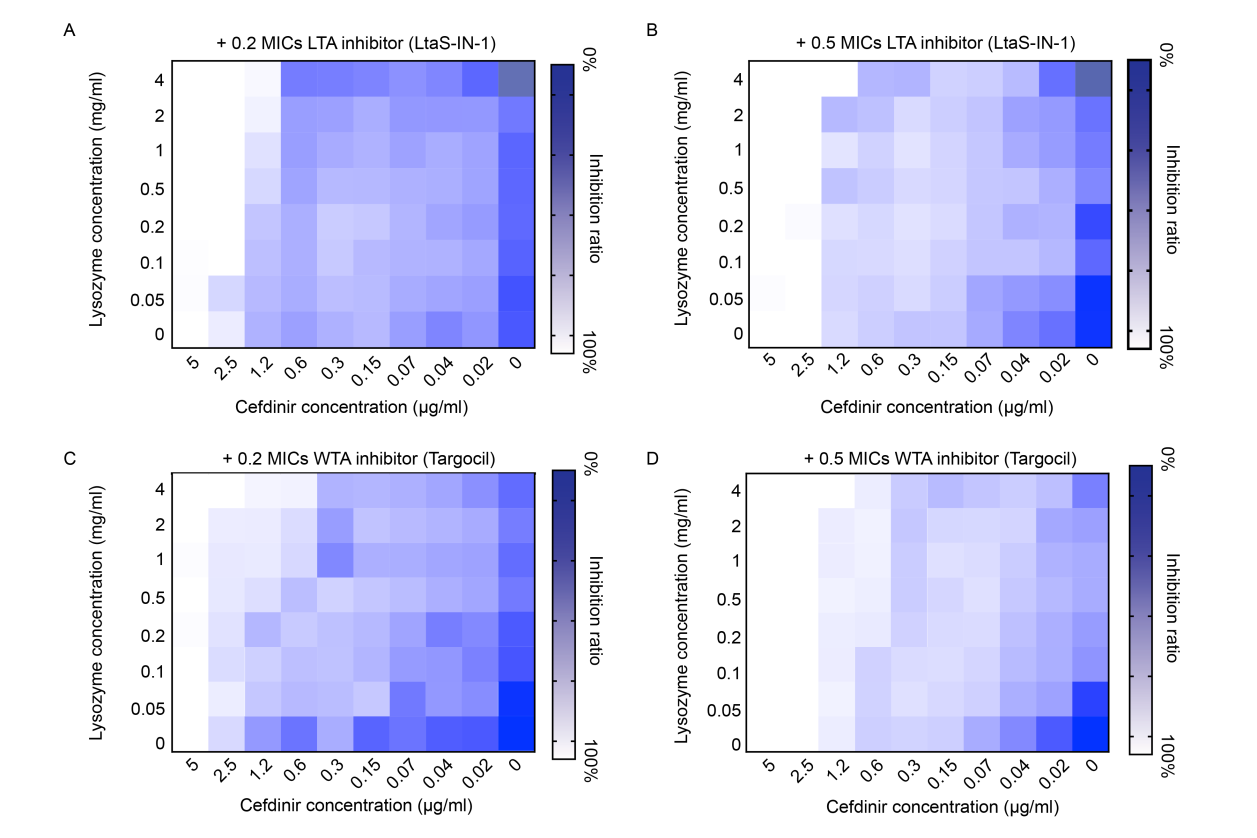


**Figure S12.** Heat maps showing the combination effects. (A-B) MRSA was cultured in LB medium containing LTA synthesis inhibitor (LtaS-IN-1) and (C-D) WTA synthesis inhibitor (Targocil). Inhibition ratios of bacterial growth were indicated by the right columns.

**
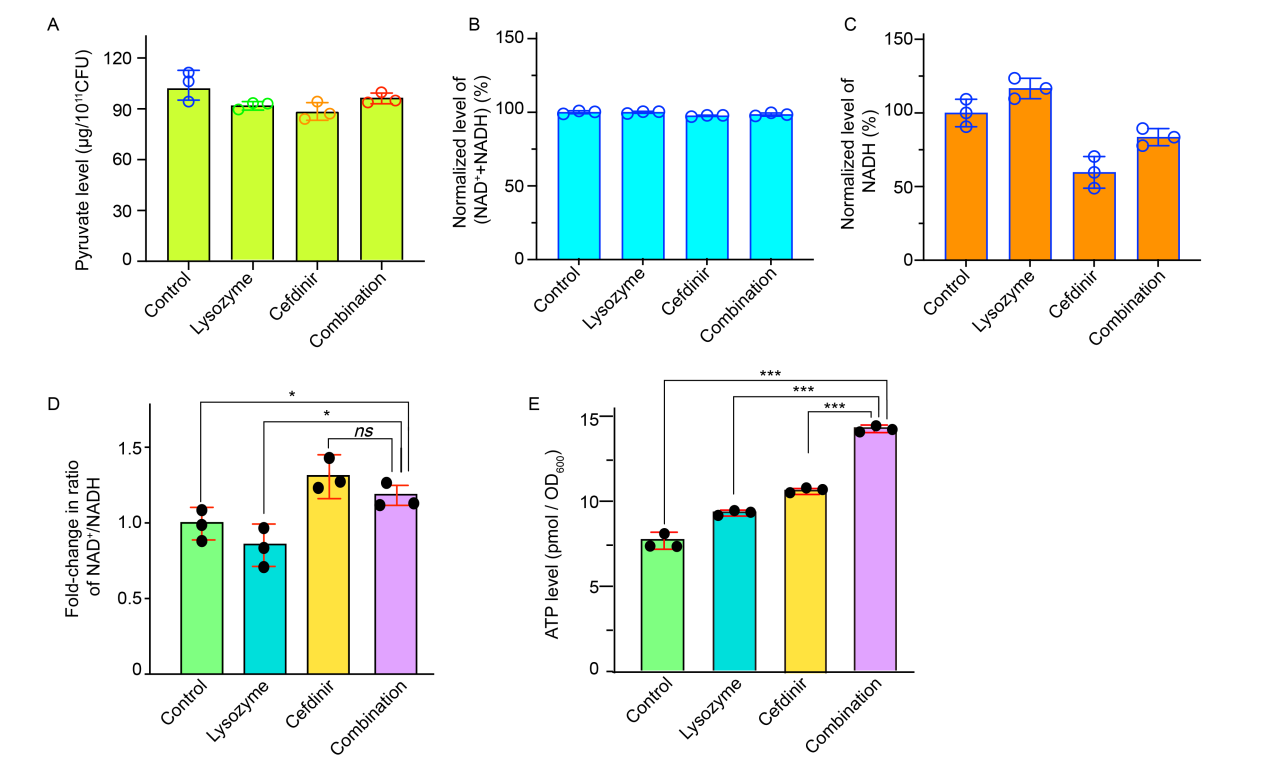
**

**Figure S13.** Combination therapy had an impact on energy metabolism. Analyses of (A) pyruvate levels, (B) total contents of NAD^+^ and NADH, (C) relative NADH levels, (D) NAD^+^/NADH ratios and (E) ATP levels in MRSA under different treatments for 1 hour. Notably, no decrease in pyruvic acid levels was observed, indicating minimal impact on upstream energy metabolism. However, NADH levels were significantly reduced in MRSA under combination treatment, leading to a 1.2-fold increase in the NAD^+^/NADH ratio. In contrast, we observed a 1.5-fold increase in ATP levels in MRSA under combination therapy, which might be partly attributed to reduced ATP consumption for cell wall synthesis, together with enhanced production mediated by the compensatory upregulation of enzymes associated with ATP synthesis in response to metabolic stress. For comparisons between two groups, the significance thresholds are set at ^*^*p*<0.05, ^**^*p*<0.01 and ^***^*p*<0.001.

**
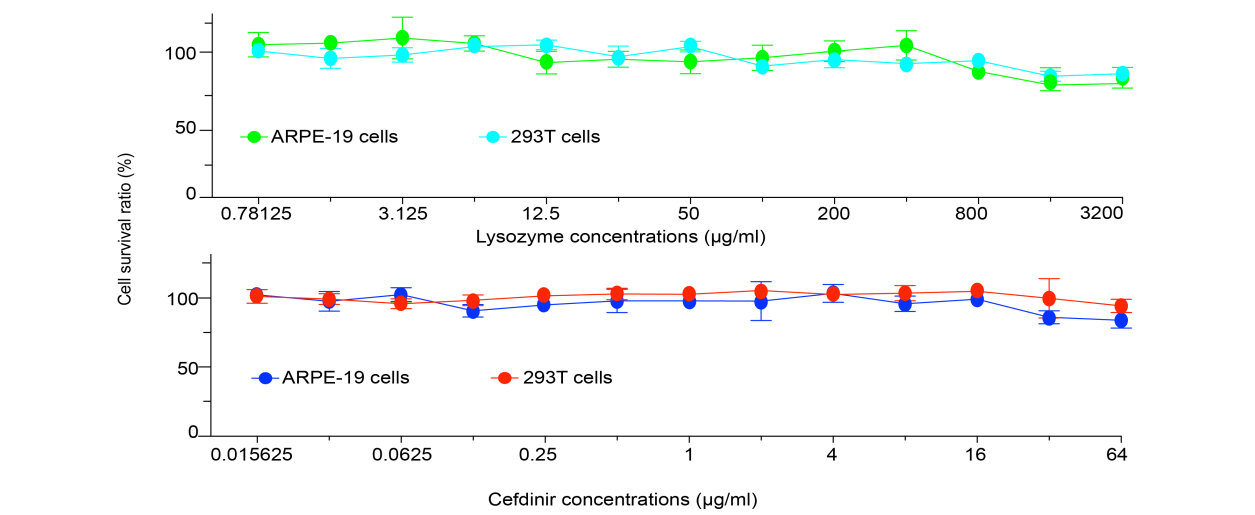
**

**Figure S14.** Relative survival ratios of ARPE-19 and 293T cells treated with lysozyme (*upper*) and cefdinir (*lower*) for 24 hours.

**
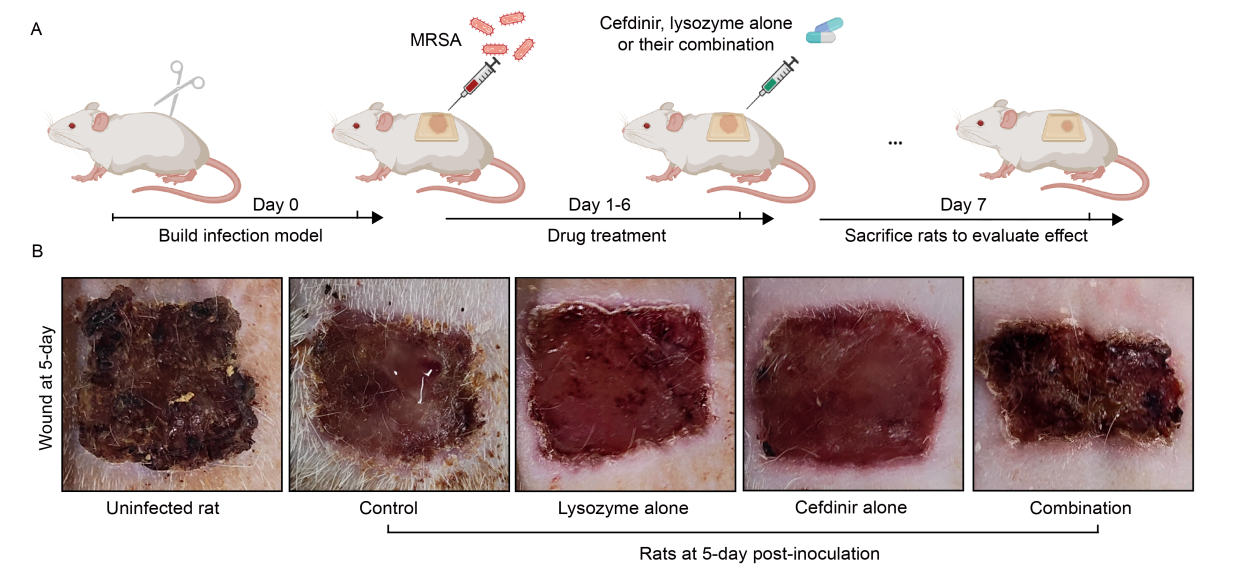
**

**Figure S15.** Combination therapy demonstrated a therapeutic effect in a rat skin infection model. (A) Flow chart showing details of the rat skin infection model. (B) Representative wound images of rats under different treatments for 5 days. Rats that received combination therapy exhibited more significant relief from scars compared to those in other groups.

**References**

1 Zhang Q, Wang R, Wang M, Liu C, Koohi-Moghadam M, Wang H, et al. Re-sensitization of *mcr* carrying multidrug resistant bacteria to colistin by silver. Proc Natl Acad Sci USA. 2022;119(11):e2119417119.

2 Sun H, Zhang Q, Wang R, Wang H, Wong Y-T, Wang M, et al. Resensitizing carbapenem- and colistin-resistant bacteria to antibiotics using auranofin. Nat Commun. 2020;11(1):5263.

3 da Silva RAG, Wong JJ, Antypas H, Choo PY, Goh K, Jolly S, et al. Mitoxantrone targets both host and bacteria to overcome vancomycin resistance in *Enterococcus faecalis*. Sci Adv. 2023;9(8):eadd9280.

4 Shen X, Yang Z, Li Z, Xiong D, Liao J, He W, et al. Identification of atypical T4SS effector proteins mediating bacterial defense. mLife. 2023;2(3):295-307.

5 Cai WJ, Lu MQ, Dai WJ. Novel antibiotic susceptibility of an RNA polymerase α-subunit mutant in *Pseudomonas aeruginosa*. J Antimicrob Chemother. 2023;78(9):2162-9.

6 Thammasirirak S, Torikata T, Takami K, Murata K, Araki T. Purification and characterization of goose type lysozyme from cassowary (*Casuarius casuarius*) egg white. Biosci Biotechnol Biochem. 2001;65(3):584-92.

7 Brott AS, Sychantha D, Clarke AJ. Assays for the enzymes catalyzing the O-acetylation of bacterial cell wall polysaccharides. In: Brockhausen I, editor. Bacterial Polysaccharides: Methods and Protocols. New York: Springer; 2019. p. 115-36.

8 Guo H, Yang Y, Zhang Q, Deng J-R, Yang Y, Li S, et al. Integrated mass spectrometry reveals celastrol as a novel catechol-O-methyltransferase inhibitor. ACS Chem Biol. 2022;17(8):2003-9.
